# Clinical Relevance of Immersive Virtual Reality in the Assessment and Treatment of Addictive Disorders: A Systematic Review and Future Perspective

**DOI:** 10.3390/jcm10163658

**Published:** 2021-08-18

**Authors:** Simon Langener, Joanne Van Der Nagel, Jeannette van Manen, Wiebren Markus, Boukje Dijkstra, Laura De Fuentes-Merillas, Randy Klaassen, Janika Heitmann, Dirk Heylen, Arnt Schellekens

**Affiliations:** 1 Human Media Interaction, University of Twente, 7522 NB Enschede, The Netherlands; j.e.l.vandernagel@utwente.nl (J.V.D.N.); r.klaassen@utwente.nl (R.K.); d.k.j.heylen@utwente.nl (D.H.); 2 Tactus Addiction Centre, 7418 ET Deventer, The Netherlands; j.vanmanen@tactus.nl; 3 Nijmegen Institute for Scientist-Practitioners in Addiction, 6525 GD Nijmegen, The Netherlands; w.markus@iriszorg.nl (W.M.); boukje.dijkstra@novadic-kentron.nl (B.D.); laura.de.fuentes@novadic-kentron.nl (L.D.F.-M.); j.heitmann@vnn.nl (J.H.); arnt.schellekens@radboudumc.nl (A.S.); 4 IrisZorg Addiction Care, 6835 HZ Arnhem, The Netherlands; 5 Radboud University Medical Centre, 6525 GC Nijmegen, The Netherlands; 6 Novadic-Kentron, Network for Addiction Treatment Service, 5261 LX Vught, The Netherlands; 7 Department of Clinical Psychology and Experimental Psychopathology, University of Groningen, 9712 CP Groningen, The Netherlands

**Keywords:** virtual reality, systematic review, addiction, assessment, treatment, cue-reactivity

## Abstract

(1) Background: Virtual reality (VR) has been investigated in a variety of psychiatric disorders, including addictive disorders (ADs); (2) Objective: This systematic review evaluates the current evidence of immersive VR (using head-mounted displays) in the clinical assessment and treatment of ADs; (3) Method: PubMed and PsycINFO were queried for publications up to November 2020; (4) Results: We screened 4519 titles, 114 abstracts and 85 full-texts, and analyzed 36 articles regarding the clinical assessment (i.e., diagnostic and prognostic value; *n* = 19) and treatment (i.e., interventions; *n* = 17) of ADs. Though most VR assessment studies (*n* = 15/19) showed associations between VR-induced cue-reactivity and clinical parameters, only two studies specified diagnostic value. VR treatment studies based on exposure therapy showed no or negative effects. However, other VR interventions like embodied and aversive learning paradigms demonstrated positive findings. The overall study quality was rather poor; (5) Conclusion: Though VR in ADs provides ecologically valid environments to induce cue-reactivity and provide new treatment paradigms, the added clinical value in assessment and therapy remains to be elucidated before VR can be applied in clinical care. Therefore, future work should investigate VR efficacy in randomized clinical trials using well-defined clinical endpoints.

## 1. Introduction

Addictive disorders (ADs), including both substance use disorders (SUDs) and behavioral addictions, are among the most prevalent psychiatric conditions with the highest global mental disease burden besides depression [1]. Globally, the prevalence of ADs varies across various substances: alcohol (4.9%), psychoactive drugs (0.2–3.5%), problematic gambling (1.5%) and tobacco use (22.5%) [2]. Though evidence-based treatments for ADs are available, these are on average only moderately effective, with around 50% relapse rates despite treatment in clinical practice [3,4]. New treatment modalities are therefore urgently needed, especially for patients that do not profit from conventional therapies.

A rather novel approach in the treatment of psychiatric disorders, including ADs, is the application of virtual reality (VR) [5]. VR is commonly described as a computer-generated simulation of a three-dimensional environment, which aims to immerse the user using special electronic equipment [6]. Typically, head-mounted displays (HMD) are used, allowing the user to feel immersed and present in a virtual environment (VE) [7]. It is thought that VR could be of great potential for both the assessment and treatment of psychiatric disorders [6,8].

VR research in the mental health field has focused predominantly on the application in anxiety disorders, such as phobias, social anxiety, post-traumatic stress disorder and obsessive-compulsive disorder, using VR exposure therapy (VRET) [9]. Through VRET, patients are systematically confronted with fear-inducing stimuli to remove the conditioned psychological response. VRET has been found to be as effective as in-vivo exposure, showing the potential of VR technology in anxiety disorders [10]. Generally, the application of VR is reported to be well-tolerated and safe in several target groups [6,11].

Various studies investigated VR in the context of ADs, mainly using cue exposure paradigms similar to anxiety disorders [6,12,13,14,15,16]. In cue exposure paradigms, patients are confronted with substance-related situations, and stimuli to elicit cue-reactivity in an ecologically valid manner [17]. Cue-reactivity refers to a conditioned response, such as subjective craving and psychophysiological responses (skin conductance, heart rate and temperature), when exposed to addiction-related stimuli [18]. The level of experienced craving during cue exposure has been linked to the severity of ADs, as well as the risk of relapse after initial abstinence [19].

Although VRET has been proven clinically effective in anxiety disorders, scientific evidence for its effectiveness in ADs is mixed [6,12]. Three recent systematic reviews summarized the evidence for VR applications in ADs [12,15,16]. Ghiţă and colleagues [15] focused on both assessment of craving and treatment possibilities with VR in alcohol use disorders. The authors conclude that there are some promising preliminary results regarding VR for both the assessment and treatment of ADs, but the study quality was found to be poor due to heterogeneity in study samples, small sample sizes and a lack of follow-up data. Trahan and colleagues [16] focused on the effectiveness of VRET in tobacco and alcohol use disorder. The authors also conclude that the number of studies is low, with limited scientific rigor.

Segawa and colleagues [12] focused on the assessment of cue-reactivity and treatment of various ADs with VR. They conclude that the VRET studies show heterogenous results and identify several methodological shortcomings, although no systematic quality assessment was applied. The authors reported positive results in provoking craving through VEs, as well as promising results of learning coping strategies as part of VR cognitive-behavioral therapy (VR-CBT). Though these interventions use VEs to expose patients to AD-related stimuli, their principles are not based on the ET paradigm described above, but rather on providing a more ecological valid environment to train these new skills.

The three systematic reviews on the application of VR in the treatment of addiction cover literature published until March 2019. Since then, several new papers with improved methodology have been published, including larger samples, a control group and the investigation of relevant clinical variables [20,21,22,23]. Previous reviews focused predominantly on the assessment of cue-reactivity. Although it has been shown that VR is a suitable environment for inducing and measuring cue-reactivity, these reviews did not address clinical correlates of VR-induced cue-reactivity, and therefore lack insight into the value for clinical use. Furthermore, several publications were not identified in the review by Segawa and colleagues [12], probably because certain databases, such as PsycINFO, were left out of the search strategy.

It is important to note that the VR field evolves rapidly, including various technological advances. All three systematic reviews on VR in ADs included studies with variable types of VR technology, including non-HMD devices. Non-HMD devices, like 3D displays with shutter glasses, are no longer regarded as immersive VR. Furthermore, the review papers cited above do not provide sufficiently detailed descriptions of the VR technical set-up. These issues limit the validity and generalizability of the conclusions in the previous reviews.

Given the limitations of previous reviews and the high speed of development in the VR field, including its application in ADs, the current review aims to evaluate the clinical relevance of VR in the assessment and treatment of ADs. To do so, we reviewed literature investigating VR-technology as a clinical assessment or intervention tool in patients with ADs, exclusively incorporating studies using an HMD. Specific research questions include: (1) What is the diagnostic/prognostic value of VR-induced cue-reactivity for the clinical assessment of patients with Ads; and (2) What is the effectiveness of VR in the treatment of patients with ADs?

## 2. Method

This systematic review was carried out in accordance with the PRISMA statement for reporting systematic reviews in healthcare [24]. We utilized the PICOS framework to formulate our research questions and identify eligible data for analysis [25].

### 2.1. Eligibility Criteria

The population considered in this systematic review were adolescents or adults with SUD, behavioral addiction or daily/heavy substance use. Only immersive VR applications that utilize an HMD for the assessment or treatment of ADs were included. Given the developmental level of the VR field in ADs, we applied rather broad inclusion criteria and as little exclusion criteria as possible (see Table 1).

### 2.2. Search Strategy

The electronic databases PubMed and PsycINFO were searched and checked by two independent authors (JH and SL) for papers published until November 2020 using the MeSH terms and keywords: (virtual) AND ((addictive) OR (addiction) OR (substance) OR (alcohol) OR (cocaine) OR (cannabis) OR (opioid) OR (tobacco) OR (nicotine) OR (methamphetamine) OR (GHB) OR (crack) OR (gaming) OR (gambling)). In addition, we conducted a backward citation search to identify articles not retrieved through the database search.

### 2.3. Study Selection

**Figure 1:**
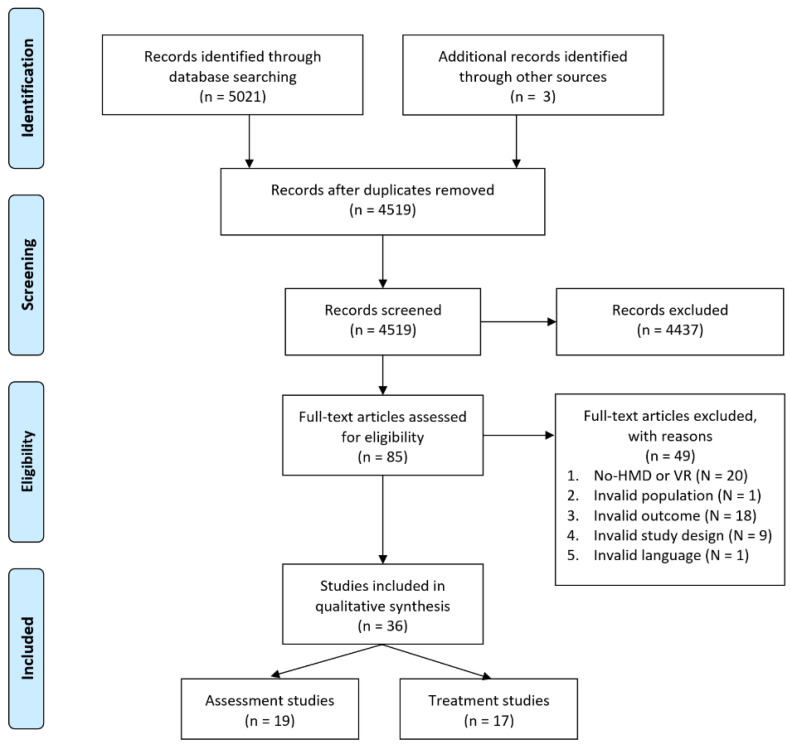
PRISMA flow diagram.

Studies were selected in three steps after conducting the literature search by following the PRISMA flow diagram (see Figure 1). All selection steps were conducted by two independent reviewers (JH, SL). First, duplicates were removed and titles were scanned based on the eligibility criteria. Afterwards, the abstracts of remaining articles were scanned to identify potentially eligible articles. In the last step, full texts of the remaining articles were scanned to exclude studies that did not meet the inclusion criteria. Any discrepancies and/or disagreements in the process between the two independent reviewers were resolved by discussion and consultation of a third reviewer (JV), where applicable. Interrater reliability was calculated for the selection steps using Cohen’s Kappa.

### 2.4. Quality Appraisal

Quality of diagnostic studies was assessed by checking whether (1) discriminative power of VR assessment was determined by means of sensitivity, specificity, predictive values, Area Under the Curve (AUC), of the Receiver Operating Characteristic (ROC) curve. If so, it was examined whether the (2) populations studied were representative for clinical populations and (3) comparison with a golden standard was performed.

Quality of effectiveness studies was assessed using the International Working Group *Recommendations for Methodology of Virtual Reality Clinical Trials in Healthcare* [26]. In this framework, VR1 studies focus on content development, VR2 studies on feasibility, acceptability, tolerability and initial clinical effects and VR3 studies on efficacy. VR3 studies provide the strongest level of evidence. VR1 studies on content development were not within the scope of this paper. Criteria for quality assessment of VR2 effect studies include representativeness of patient population, sample size, selection of clinically relevant Patient-Reported Outcome measures and pre-post measurements [26]. Quality of VR3 studies was assessed based on (1) representativeness of the population, (2) use of an empirically validated treatment comparison, (3) follow up of clinical outcomes, (4) sample size and power and (5) use of randomization and/or a control group.

### 2.5. Data Extraction

The data extraction template was developed based on the Cochrane data extraction sheet for intervention reviews and pilot-tested prior to data extraction [27]. Data were extracted by five reviewers (LDFM, JvM, BD, WM, JV) and checked for accuracy and completeness by a second reviewer (SL). The following information was extracted for each study: (a) publication (author(s), year, country of origin, publication type), (b) methods (aim of study, duration of study, study design), (c) participants (e.g., sample size, control group, dependence severity, age, gender, ethnicity, comorbidities, inclusion/exclusion criteria, (d) assessment/treatment (procedure, setting, provider information, comparators, assessment instrument, follow-up, time-points measured, cues in environment, multisensory, technological aspects), (e) outcome measures of interest (clinical outcomes and secondary outcomes, response-rate, drop-outs, and (f) risk of bias and study quality. In case papers were inconclusive regarding methods and results, the corresponding authors were contacted to elucidate the issues.

### 2.6. Data Synthesis

A narrative approach was used to synthesize the findings because of the heterogeneity in terms of study design, methods, assessment and treatment approaches, as well as (clinical) outcome measures (Table 2, Table 3 and Table 4). The results are summarized, describing and explaining the study characteristics and outcomes in text and tables. In the result section, the findings based on this data synthesis were separately described for assessment and treatment studies. A table with definitions and descriptions of concepts in VR in general, VR technology, cue-reactivity in VEs, cues in VEs, and VR treatment approaches can be found in the supplementary materials (Table S1 in Supplementary Materials). 

## 3. Results

### 3.1. Study Selection

The electronic database search (Figure 1) identified 5021 records of interest. After removing the duplicates, 4519 records remained, which were screened for eligibility. After screening for title and abstract, 4437 studies were excluded. Finally, 82 full-text articles were assessed for eligibility. Furthermore, three additional resources were identified through the backward citation search for eligible papers, and 49 papers were excluded because the studies did not use immersive VR through HMDs (*n* = 20), ineligible outcomes were reported (*n* = 18), an ineligible study design (case studies, study protocols) was employed (*n* = 9), population without AD or daily/heavy use (*n* = 1), or the paper was written in a different language than English (*n* = 1). Cohen’s Kappa for the title and abstract screening (eligible, ineligible, maybe) was substantial (*K* = 0.65, *CI* = 0.59–0.71; *K* = 0.62, CI = 0.50–0.75, respectively). A total of 36 studies were included in the review and divided into assessment (*n* = 19) and treatment (*n* = 17) studies.

### 3.2. Clinical Relevance of VR in the Assessment of ADs

#### 3.2.1. General Description of the Included Studies

We identified 19 papers presenting relevant findings toward VR as a tool in the clinical assessment of ADs (Table 2). All studies reported a relation between cue-reactivity and one or more clinical parameters, thereby providing insight in the diagnostic value of VR-induced cue-reactivity (in assessing e.g., addiction severity). However, two studies specifically analyzed the discriminative power (e.g., sensitivity, specificity, predictive values), and are therefore considered the most informative regarding the diagnostic possibilities of VR in ADs [23,28].

All publications assessed the ability of (multiple) VEs (substance-related and or neutral) to induce cue-reactivity using a single session (*n* = 17) or more sessions (*n* = 2). Studies reported single-group (*n* = 7) and between-group (*n* = 14) designs with moderate sample sizes (*n* = 11–665; median = 40).

Participants used tobacco (*n* = 8), alcohol (*n* = 7), methamphetamine (*n* = 3), or were gaming participants (*n* = 1). The reported mean age of subjects ranged from 18 to 43 (m = 31.8), and men (range = 18–100%, m = 63%) were more represented than female. AD criteria were reported in 10 studies, using the screening instruments Fagerström Test for Nicotine Dependence (FTND, *n* = 4) and Cigarette Dependence Scale for nicotine dependence (CDS, *n* = 1), as well as Alcohol Use Disorders Identification Test (AUDIT, *n* = 3) and Alcohol Dependence Scale (ADS, *n* = 2) for alcohol dependence. Units per day/week/month were reported in 13 studies. The mean AD severity was heterogeneous across studies.

The VEs varied greatly regarding several aspects, including cues utilized (proximal, contextual and complex) and the exposure time in the VE (3–150 min). Multisensory VEs were utilized using visual, auditory, olfactory and haptics (*n* = 2), visual, auditory and olfactory (*n* = 4), visual and auditory (*n* = 12) stimuli, while possibilities to interact with the VE were reported in 12 papers. The VEs were presented using an HMD (*n* = 18) vs. smartphone HMD (*n* = 1) showing computer-generated (*n* = 16) or 360-degree videos (*n* = 3).

#### 3.2.2. Clinical Assessment

Most publications (*n* = 17) assessed cue-reactivity by means of craving and some by means of heart rate variability (HRV, *n* = 2), skin conductance (*n* = 2), anxiety (*n* = 2), temperature (*n* = 1), withdrawal (*n* = 1), attention toward cues (*n* = 1) and thinking about using a substance (*n* = 2). The assessment instruments were self-report measures, mostly visual analogue scales (*n* = 24) and questionnaires (*n* = 7, e.g., QSU-Brief). Five studies used other measures, namely skin conductance, temperature, ECG and EEG.

Clinical disease status was indicated by level of dependence (*n* = 9), level of substance use (*n* = 6), treatment seeking (*n* = 3), level of withdrawal (*n* = 1), CO levels (*n* = 1) and anxiety (*n* = 1). If groups were compared, comparisons were made between subgroups (e.g., (heavy) users versus light/nonusers; *n* = 5), patients with dependence versus non-dependence (*n* = 5), treatment seekers versus non-treatment seekers (*n* = 1) and between different treatment conditions (*n* = 1).

#### 3.2.3. VR Findings

Most studies (15/19) reported one or more significant associations of VR-cue-reactivity with clinical parameters (see Table 2). Two-thirds of the studies that showed significant associations reported the strength of the associations (*n* = 10). Eight indicated moderate to strong associations between a single cue-reactivity parameter and clinical status. Correlation coefficients ranged from r = 0.45–0.78 (*n* = 4) and effect sizes from d = 0.35–1.46 (*n* = 4).

Two studies reported statistical parameters on the discriminative power of cue-reactivity measures [23,28]. These two studies combined multiple cue-reactivity parameters –based on ECG (HRV), EEG, and/or skin response (GSR)—into one or more classifiers. In the study of Ding et al. [23], the AUC value ranged from 0.95–0.97, indicating excellent discrimination between methamphetamine patients and healthy controls. In the study of Wang et al. [28], the best classifier showed positive and negative predictive values of 90% and 83% respectively when discriminating between methamphetamine-dependent patients and healthy controls.

Two studies that used the FTND to assess the level of tobacco dependence found significant correlations with cue-reactivity. Most studies on alcohol that used VR-induced craving (5/7) found a significant correlation with clinical status. One study evaluating cue-reactivity in VR versus smartphone found positive results in favor of smartphone use.

#### 3.2.4. Quality of the Studies

VR techniques and procedures highly varied between studies. Comparisons between different techniques or procedures within studies were not made, thereby limiting the ability to make further design decisions based on these studies. Only the studies of Wang et al. and Ding et al. [23,28] tested the discriminative or diagnostic value of VR-induced cue-reactivity (for methamphetamine addiction). However, the included study populations were not representative for the general population, considering the prior probability of substance use, which will be much lower than 42–50%.

**Table 2:** Virtual reality assessment studies.

Reference	Design	Population	VR Assessment	Measurements		Clinical Outcome *
				Indicator of Clinical Status	Indicator of Cue-Reactivity	
**Nicotine studies**						
Ferrer-Garcia et al. (2010) [29]	Within-group correlation	- NTS former smokers, cigarettes/day = 14.8 (*n* = 25)	- Computer-generated, multisensory (visual + auditory), complex VEs with agents: (1) being in a pub, (2) having lunch at home, (3) having breakfast at home, (4) drinking coffee in a cafe, (5) after lunch at restaurant, (6) waiting in the street, (7) watching TV at night, (8) neutral museum - Interaction: with agents + objects via mouse device - Minutes/VE: unknown	- Cigarettes/day	- Craving ^a^	- n.s.
Ferrer-Garcia et al. (2012) [30]	Within-group correlation	- NTS, cigarettes/day = 15.6, FTND = 3.4 (*n* = 46)	- As in Garcia-Rodriguez et al. (2012) - Interaction: with agents + objects via mouse device - 6 min/VE	- Dependence ^b^ - Cigarettes/day - CO levels - Anxiety ^c^	- Craving ^a^	- Positive prediction (R2 = 0.25, *p* ≤ 0.01) in 1 VE (breakfast at home) - n.s. - n.s. - n.s.
Garcia-Rodriguez et al. (2012) [31]	Between groups comparison	- Smokers, cigarettes/day = 15.6 (*n* = 46) - Never smokers (*n* = 44)	- Computer-generated, multisensory (visual + auditory), complex VEs with agents: (1) being in a pub, (2) having lunch at home, (3) having breakfast at home, (4) drinking coffee in a cafe, (5) after lunch at restaurant, (6) waiting in the street, (7) watching TV at night, (8) neutral museum - Interaction: with agents + objects via mouse device - 6 min/VE	- Smokers vs. no smokers	- Craving ^a^ - Heart rate - Temperature - Skin resistance	- n.s. - smokers > non-smokers (5 VEs, d = 0.48–0.63) - smokers > non-smokers (2 VEs, d = 0.50–0.52) - n.s.
Bordnick et al. (2013) [32]	Between groups comparison	- TS, cigarettes/day = 23.8 (*n* = 82) - NTS smokers, cigarettes/day = 25.4 (*n* = 23)	- Computer-generated, multisensory (visual, audio, haptics + olfactory), proximal + complex VEs with agents: (1) neutral office interview, (2) paraphernalia room, (3) party room - Interaction: no/limited - 3 min/VE	- Treatment seekers vs. nontreatment seekers	- Craving ^a^	- n.s.
Pericot-Valverde et al. (2013) [33]	Within-group (pre-post abstinence) comparison	- Smokers willing to quit, cigarettes/day = 14.7, FTND = 3.1, NDSS = 55.2 (*n* = 11)	- As in Garcia-Rodriguez et al. (2012) - Interaction: with agents + objects via mouse device - Before abstinence, 24 h after abstinence, 7d after abstinence - Max. 30 min	- CO levels - Cigarettes/day	- Craving ^d^	- Before abstinence > 24 h and 7d of abstinence (*p* = 0.03)
Thompson-Lake et al. (2015) [34]	Within-group correlation	- NTS deprived from smoking overnight, cigarettes/day = 18.3, FTND = 6.0, (*n* = 36)	- As in Bordnick et al. (2013), without haptics - Interaction: no/limited - 3 min/VE	- Dependence ^b^ - Withdrawal ^e,f^	- Craving ^a^	- r = 0.58, *p* < 0.01 - n.s.
De Bruijn et al. (2020) [35]	Between groups comparison Subanalyses within a randomized experiment	- Smokers, CDS = 2.9–3.0 (*n* = 58) - Recent quitters, CDS = 1.3–1.5 (*n* = 30)	- 360-degree video, multisensory (visual + auditory), complex VEs (low vs. high immersion): (1) being in a pub, (2) waiting in the street on public transport to arrive, (3) in the morning after having breakfast, (4) neutral video - No interaction: exposure - Randomization of participants to smartphone/VR headset condition - 2:30 min/VE	- Current smokers vs. recent quitters	- Craving ^a,g^	- Craving ^a,g^ VR: n.s. - Craving ^a^ smartphone: smokers > recent quitters (*p* < 0.01, n^2^ = 0.11) - Craving ^a^: n.s.
Kotlyar et al. (2020) [36]	Between groups comparison Subanalyses within a randomized cross-over study	- Smokers deprived from smoking overnight, cigarettes/day = 16.8, FTND = 5.3, (*n* = 58)	- As in Bordnick et al. (2013), without olfactory + haptics - Interaction: no/limited - 3 sessions: no treatment, nicotine lozenge, placebo lozenge - 3 min/VE	- No treatment, nicotine lozenge, placebo lozenge	- Craving ^a^ - Craving ^h^ - Urges ^i^ - Withdrawal ^h^ - Thinking about smoking ^a^	- During VR and neutral cues (not after): - placebo lozenge (ES = 0.52, *p* < 0.01) and nicotine lozenge (ES = 0.72, *p* < 0.01) < no treatment - placebo lozenge (ES = 0.35, *p* = 0.01) and nicotine lozenge (ES = 0.60, *p* < 0.01) < no treatment - placebo lozenge (ES = 0.48, *p* < 0.01) and nicotine lozenge (ES = 0.70, *p* < 0.01) < no treatment - placebo lozenge (ES = 0.36, *p* < 0.01) and nicotine lozenge (ES = 0.37, *p* < 0.01) < no treatment - placebo lozenge (ES = 0.41, *p* < 0.01) and nicotine lozenge (ES = 0.47, *p* < 0.01) < no treatment - Placebo lozenge vs. nicotine lozenge: n.s.
**Alcohol studies**						
Bordnick et al. (2008) [37]	Within-group correlation	- NTS AUD (*n* = 33) - NTS alcohol abuse (*n* = 7) - Units/day = 5.1	- Computer-generated, multisensory (visual, auditory, olfactory-scent Palette system- + haptics), complex VEs with agents: (1) neutral aquarium scene, (2) bar cue, (3) kitchen, (4) argument environment, (5) party - No interaction: exposure - 3 min/VE (18 min)	- Drinks/day (categorical) - Smoking/non-smoking	- Craving ^a^	- n.s. - n.s.
Lee et al. (2008) [38]	Between groups comparison	- Abstinent male AUD patients, 63.1 units/week, ADS = 22.1 (*n* = 14) - Age-matched social drinkers, 1.4 units/week, ADS = 3.1 (*n* = 14)	- Computer-generated (+360-degree background), multisensory (visual + auditory), complex VE with agent: (1) neutral virtual street, (2) virtual pub with alcohol cues - No interaction: exposure - 4 blocks alcohol cues (ALC) and social pressure (SP): −ALC & −SP, −ALC & +SP, +ALC & −SP, +ALC & +SP - Min/VR not reported	- AUD vs. social users (SU)	- Craving ^a^	- Craving VE: AUD > SU - Craving social pressure: AUD = SU (n.s.) - Group × Alc-VE × Social pressure: interaction effect (*p* < 0.01)
Ryan et al. (2010) [39]	Between groups comparison	- Bingers college students, 8.1 units/sitting (*n* = 15) - Non-bingers college students, 0.6 units/sitting (*n* = 8)	- Computer-generated, multisensory (visual, auditory + olfactory-scent of a favorite drink-), complex + personalized proximal VEs with agents: (1) neutral aquarium scene, (2) bar cue, (3) kitchen, (4) argument, (5) party - No interaction: exposure - 3 min/VE	- Bingers (B) vs. non-bingers (NB)	- Craving ^a^ - Amount of attention to the sight and smell of alcohol ^a^ - Thinking of drinking ^a^	- B > NB: kitchen, party rooms drinkers (*p* = 0.02), barroom, argument room, neutral (n.s.) - n.s. - B > NB: bar and party rooms (*p* = 0.04), VR kitchen (n.s.), argument (n.s.) & 2 neutral rooms (n.s).
Traylor et al. (2011) [40]	Between groups comparison	- NTS smokers & AUD, 6.9 units/day, ADS = 16.5 (*n* = 14) - NTS smokers & ND daily drinkers, 3.9 units/day, ADS = 5.9, (*n* = 7)	- Computer-generated, multisensory (visual, auditory, olfactory), complex VEs with agents: (1) neutral video, (2) party, (3) office - No interaction: exposure - 3 min/VR	- AUD vs. social users (SU) ^j^	- Craving ^a^ alcohol - Craving ^a^ nicotine	- Alcohol craving VR/neutral context: AUD > SU (*p* < 0.01): office scene (d = 1.46, *p* < 0.01), 2nd neutral (d = 1.43, *p* < 0.01), party scene (n.s.), 1st neutral (n.s.) - Alcohol craving X VR/neutral: AUD = SU (n.s.) - Nicotine craving between groups & interaction: n.s.
Ghita et al. (2017) [41]	Between groups comparison	- Light drinkers (LD) college students, 2.9 units/month, AUDIT = 2.8 (*n* = 13) - Heavy drinkers (HD) college students, 23.5 units/month, AUDIT = 7.3 (*n* = 12)	- Computer-generated, visual -only (participant choose personalized alcoholic or non-alcoholic drink, complex VEs: (1) restaurant, (2) bar, (3) bedroom, (4) chill-out area - Interaction: limited (joystick locomotion) - Min. 10 sec/VE	- Heavy users (HU) vs. light users (LU) - Dependence ^k^	- Craving ^a^ alcohol - Anxiety ^a^ - Choosing between alcoholic or non-alcoholic cues	- n.s. - n.s. - HU > LU (*p* < 0.05): restaurant/bar (*p* < 0.05), chill-out & bedroom (n.s.)
Ghita et al. (2019) [20]	Between groups comparison and within group correlation	- AUD outpatients, AUDIT = 23.8 (*n* = 13) - Social drinking students, 9.2 units/month, AUDIT = 4.5 (*n* = 14)	- Computer-generated, multisensory (visual, auditory + olfactory, proximal + personalized drinks), complex VEs with agents: (1) restaurant, (2) bar, (3) pub, (4) at-home environment, (5) neutral with glass of water - Interaction: full body interaction with agents + environments (incl. grabbing with Oculus touch controllers) - 10–15 min/VE, 1 h total	- AUD vs. social users (SU) - Dependency symptoms ^k^	- Craving ^a^ alcohol - Anxiety ^a^	- Craving: AUD > SU (*p* < 0.05) - Anxiety: AUD > SU (*p* < 0.05) - AUD: - Craving related to dependency symptoms (r = 0.78, *p* < 0.01) - Anxiety related to dependency symptoms (r = 0.65, *p* = 0.02) - SU: - Craving related to dependency symptoms: n.s. - Anxiety related to dependency symptoms: (r = 0.7, *p* = 0.0005)
Simon et al. (2020) [42]	Between groups comparison	- Heavy drinkers, AUDIT = 15.2 (*n* = 18) - Occasional drinkers, AUDIT = 3.8 (*n* = 21)	- Computer-generated, multisensory (visual + auditory), complex VE with agents: a visual bar - Interaction: limited (joystick locomotion) - 2–3 min/VR space, 8–10 min total	- Heavy users (HU) vs. occasional users (OU) ^k^	- Craving ^a^	- HD > OU (*p* < 0.01, d = 1.00)
**Methamphetamine studies**						
Wang et al. (2018) [28]	Between groups comparison	- MD male after MA detox (*n* = 61) - Age-matched male healthy controls (*n* = 45)	- 360-degree video, multisensory (visual + auditory), complex VEs: (1) voice with black screen to take drugs, (2) person playing with “ice” and drug paraphernalia, (3) first-person perspective observing people using meth, (4) close-up people using meth (facial expression), (5) meth use, (6) prepared drug and related paraphernalia presented and moved toward participant - No interaction: exposure - Equipment (i.e., heart rate recording device, VR helmet and headphone) - 8 min video	- Abstinent patients with MD vs. healthy controls (HC)	- sup>- Craving ^a^ - Heart rate ^l^	- Discriminant Model cue-induce ECG algorithm: high predictive power distinguishing MD from HC (*p* < 0.001, classification accuracies: sensitivity 86.9%, specificity 86.7%, PPV 89.8%, NPV 83.0%)
Tan et al. (2019) [43]	Between groups comparison	- MD inpatients, days of use last month = 10.6 (*n* = 60); HU and LU, use day last month (*n* = unknown)	- 360-degree video, multisensory (visual + auditory), complex VE: (1) living room in private house, prepare ice-pot, smoke with satisfied expression, (2) neutral beach - No interaction (exposure) - 5 min per cue environment	- Heavy users (HU) vs. light users (LU)	- Craving ^a^ - Heart rate ^l^ - SCL	- n.s. - n.s. - n.s.
Ding et al. (2020) [23]	Between groups comparison	- Abstinent inpatient male MD, MA use = 65.6 months (*n* = 333) - Age-matched male healthy controls (*n* = 332)	- Computer-generated, multisensory (visual + auditory), complex VEs with agents: (1) neutral cue, (2) karaoke, (3) bedroom, (4) car, all with meth cues - Interaction: full-body interaction with grabbing and using objects - 4 min/VE	- MD vs. healthy controls (HC)	- Brain activities ^m^ - Skin response ^o^	- GSR, EEG power (delta/alpha bands): MD < HC (*p* < 0.01) - EEG power (beta/gamma band: MD > HC (*p* < 0.001) - EEG power (theta): n.s. - EEG & GSR combined: AUC 0.95–0.97
**Gaming studies**						
Shin et al. (2018) [44]	Between groups comparison and within group correlation	- Male adolescents & young adults IGD, hours gaming/week = 27.9, IAT = 55.5 (*n* = 34) - Healthy controls, hours gaming/week = 5.2, IAT = 35.1 (*n* = 30)	- Computer-generated, multisensory (visual + auditory), complex VEs with agents: 4 VR tasks in internet café: (1) entrance, (2) conversation observation, (3) gaming invitation; (4) refusal skills practice. - Interaction: Full body interaction with leap-motion device - Min/VR not reported	- IGD vs. healthy controls (HC) - Dependence ^p^	- Craving ^a^	- Craving internet café: IGD > HC (*p* < 0.001) - IGD group: positive correlation craving and dependence severity (r = 0.446, *p* = 0.008 entering café

* association between disease severity and cue-reactivity. Abbreviations: AUD = Alcohol Use Disorder; AUDIT = Alcohol Use Disorder Identification Test; CDS = Cigarette Dependence Scale; CO = Carbon monoxide; ECG = Electrocardiogram; FTND = Fagerstrom Test of Nicotine Dependence; IGD = Internet Gaming Disorder; IAT = Alcohol-Implicit Association Task; MA = Methamphetamine; MD = Methamphetamine Dependent (according to DSM-IV criteria (Diagnostic and Statistical Manual of Mental Disorders, 4th edition); ND = Nicotine Dependent; NTS = Non-Treatment Seeking; TS = Treatment Seeking; VE = Virtual Environment; HU = Heavy Users; LU = Light Users; SU = Social Users; OU = Occasional Users; B = Bingers; NB = Non-Bingers; HC = Healthy Controls. Measurement instruments: ^a^ Visual Analogue Scale (VAS), ^b^ Fagerstrom Test of Nicotine Dependence (FTND), ^c^ State-Trait Anxiety Inventory (STAI), ^d^ Peak provoked craving, ^e^ Wisconsin smoking withdrawal scale, ^f^ Shiffman-Jarvik withdrawal questionnaire, ^g^ Short form of the tobacco Craving Questionnaire, ^h^ Minnesota Nicotine Withdrawal Scale (MNWS), ^i^ Questionnaire of Smoking Urges (QSU), ^j^ Alcohol Dependence Scale (ADS), ^k^ Alcohol Use Disorders Identification Test (AUDIT), ^l^ ElektroCardioGram, ^m^ Electroencephalography, ^o^ Galvanic Skin Response, ^p^ Young’s Internet Addiction Test.

### 3.3. Clinical Relevance of VR in the Treatment of ADs

#### 3.3.1. General Description of the Studies

We identified 17 papers, based on 19 studies, presenting clinical outcomes after a VR intervention (Table 3 and Table 4). Most were VR2 studies [26]. Two papers described results from multiple studies [22,45], and several papers described data of the same group, e.g., Pericot-Valverde et al. [46,47,48,49].

Study subjects ranged from 17.0 to 63.4 in age and were predominantly male (*n* = 1121, 67% of the total known sample). Four studies only included men [22,50,51,52], one did not report gender [53]. Most studies reported AD criteria (*n* = 15), based on DSM-IV, DSM-IV-TR or DSM-5, or specific assessment instruments (e.g., AUDIT, FTND). AD severity ranged from moderate (*n* = 9) to high (*n* = 6). Most participants were treatment-seeking (*n* = 11). Some studies used non-treatment seeking samples (*n* = 6) or abstaining participants (*n* = 2).

#### 3.3.2. Treatment Studies Using a VR Exposure Therapy Paradigm

About half of the studies (*n* = 10/19) used a VRET approach (Table 3). Half of these used VRET as a stand-alone intervention, the rest used it as an add-on to another intervention (e.g., CBT or mindfulness). Most VRET studies (*n* = 9/10) provided multiple VRET sessions (range = 5–15, each lasting 20–50 min). VRET studies focused on tobacco use (*n* = 8), alcohol use (*n* = 1) and gambling (*n* = 1).

In the VRET studies, participants were exposed to a combination of discrete or proximal cues (e.g., a lighter or cigarette) in a typical VE (e.g., a café). Mostly, the user could interact with objects or agents in the VE (e.g., refusing a cigarette when offered). The VEs were mostly visual and auditory (*n* = 8) supplemented with olfactory or haptics (*n* = 2). All VEs were presented using an HMD, showing computer-generated environments (*n* = 9) or 360-degree videos (*n* = 1). All VRET studies used either craving or urge to gamble as (one of the) outcome measurements.

Four of the ten VRET studies found null results at all [51,52,54,55], while two reported negative effects of VRET [47,49]. Five studies found a reduction in craving after VRET [21,46,48,49,56]. Only three of these studies reported effect sizes (ηp^2^ = 0.47–0.76 or d = 0.44). Furthermore, Pericot-Valverde et al. [47] reported a reduction in cravings, but did not report whether this was significant.

Studies using substance use as an outcome measure (*n* = 6/10) showed positive effects of VRET in two studies [46,56]. Only Pericot-Valverde et al. [46] reported effect sizes regarding number of cigarettes per day and air expired CO levels (ηp^2^ respectively 0.82 and 0.49). Two studies found no effect on substance use [49,52]. Lee et al. [51] found a reduction of cigarettes smoked during the morning but not on a daily basis. Finally, two studies showed negative effects of VRET on substance use [47,49].

Studies using other addiction-related variables generally showed no effects of VRET on severity scores of dependence or withdrawal [47,51,55,56]. One study found that readiness to quit increased in smokers allocated to the VRET group, compared to a control group [56]. However, the VRET group in this study also received mindfulness, peer-to-peer and conditional support, while the control group only received a smoking cessation manual without any support. Giroux et al. [54] found no change in perceived self-efficacy in gamblers.

Finally, four studies reported on effects of VRET on treatment retention, with two showing positive effects [21,56] and two showing no clear effect [49,55]. Of note, Goldenhersch et al. [56] reported a very high number of completers (93%) in the experimental condition, which they attributed to the use of strategies to enhance adherence, such as SMS text messaging and phone call reminders.

#### 3.3.3. Treatment Studies Using Other VR Paradigms

The other studies (nine studies, reported in seven papers) used a variety of treatment paradigms other than VRET (Table 4). These studies focused on tobacco use (*n* = 3), alcohol use (*n* = 2), methamphetamine use (*n* = 2) and gambling (*n* = 2). In five of these studies, the VR intervention was a stand-alone intervention, in four VR was provided as an add-on to another intervention (e.g., CBT). Most studies (*n* = 7) provided multiple VR sessions (range = 2–10, each lasting 6–60 min) and two studies used a single VR session [50,57].

Participants were exposed to a combination of proximal cues, in a fitting contextual VE and mostly (six of the studies) complex VEs, in which the user could interact with objects or agents in the VE. Only two studies applied a passive paradigm [50,58]. Most VEs included multisensory cues (mostly auditory (*n* = 4) or auditory, olfactory and/or haptics (*n* = 2)). Studies used computer-generated (*n* = 6) or 360-degree videos (*n* = 1).

Most non-VRET studies (*n* = 8) used several different VEs (range 2–6) to expose the participants to multiple ecologically valid VEs. None of the studies used an individualized hierarchy. One study applied a generic hierarchy in a virtual bar or casino and guided participants progressively, approaching machines where they could gamble whilst applying various CBT techniques at each step [45].

Three studies used complex aversive stimuli (e.g., scenes of vomiting in the subway, police arrest, substance use-related illness) paired with nicotine, alcohol or methamphetamine use, respectively, to motivate participants to reduce unwanted behavior (aversive learning) [22,50,59]. Girard et al. [53] instructed participants in the experimental group to find and crush up to 60 virtual cigarettes in a VE. In contrast, control participants crushed balls instead. We categorized this approach under the term ‘embodied learning’. Two studies used VR to train coping skills to deal with respectively nicotine craving and gambling urges in a CBT framework, with gradually increasing difficulty [45,58].

Finally, one study used VR to assess drinking behavior, psychological factors (emotion regulation and self-esteem) and social factors (relational competence and social pressure on drinking behavior) [57]. During immersion, the researcher would ask questions like: “Imagine you have just drunk a glass of wine, how do you feel?; Would you call anyone from your family?”, in order to evoke coping-related imagery, negative memories of a relapse or increase motivational status.

About half of the studies (*n* = 4/9) used craving or urge to gamble as (one of the) outcome measurements (e.g., QSU-brief, VAS, Alcohol Urge Questionnaire, Gambling Craving Scale). Self-reported substance use was measured in two of the non-VRET nicotine studies [53,58], with biomarker confirmation (exhaled carbon monoxide) in one study [53]. Most non-VRET papers reported other addiction-related variables, like severity scores [45,53], motivation [59] or readiness to change [57], self-efficacy and confidence [58], gambling-related cognitions [45], psychophysiological measures (HRV) [22] and implicit cognitions [50].

Studies using craving as outcome measure generally found positive effects of the non-VRET intervention (*n* = 3/4), for nicotine, alcohol and methamphetamine, respectively [22,50,58]. Only Bordnick et al. [58] reported a large effect size (ηp^2^ = 0.37). The article on gamblers found no beneficial effects of the VR intervention [45].

Studies using substance use (both tobacco) as outcome measure showed positive effects of the VR intervention in terms of abstinence rates, confirmed by CO measures [53] and the mean number of cigarettes used per day or week at one, two- and six-months follow-up [58]. Interestingly the effect of VR seemed to have increased over time [53], however low retention hampers strong conclusions. Furthermore, Bordnick et al. [58] reported a large effect size of ηp^2^ = 0.14.

The eight studies reporting other addiction-related variables were generally positive, but showed some mixed findings. One study showed positive effects of the VR intervention on nicotine dependence level compared to a control condition [53], but this was not observed in gamblers [45]. Caponnetto et al. [59] found beneficial effects of the active VR intervention on motivation to quit smoking, compared to a passive image or video. Similarly, Spagnoli et al. [57] found beneficial effects of the VR interventions on the readiness to quit alcohol use, compared to those receiving regular care.

Self-efficacy and confidence to resist smoking increased respectively post-intervention and at follow-up 1, 2, 3 and 6 months, compared to the control condition, with medium to large effect size (ηp^2^ = 0.13) [58]. In contrast, gambling-related cognitions were not influenced by the VR intervention in a group of gamblers, compared to the control condition [45]. The one study using HRV reported a significant decrease of several—yet not all—indexes, suggesting that the VR sensitization procedure suppressed cue-induced reactivity in methamphetamine users [22]. Similarly, reductions in implicit alcohol associations were observed after a VR intervention in both high and low social drinkers (η^2^ = 0.14) [50].

Of note is that drop-out was a major issue in several non-VRET studies. Girard et al. [53] found higher drop-out rates during the control condition, compared to the intervention (49% vs. 22%) and at the end of the 12-week program (71% vs. 50%). Bordnick et al. [58] also reported substantial drop-out in both conditions before (17% vs. 18%) and during treatment (29% vs. 42%). Caponnetto et al. [59] experienced no drop-out, while Wang et al. [22] did report a loss-to-follow up, without further analysis. Some studies, including those using a single VR session [50,57], did not report retention [45].

#### 3.3.4. Quality of the Treatment Studies

The majority of the included intervention papers (*n* = 14/17) could be regarded as developmental studies (VR2). Only one study was a clear efficacy study (VR3) [49]. Two studies seemed to be intended as VR3 studies, but provided only preliminary evidence for efficacy, due to limited number on inclusions (lack of power) in comparison with the original protocol publication [55,60], or reporting of pilot data only, without further power analysis or availability of a comparison group [53].

Six papers included either addiction severity or addictive behavior as outcome measure, while three used both, and eight lacked information on addiction severity or addictive behavior. Furthermore, five of the nicotine papers and one of the gambling papers used non-treatment-seeking participants that seemed to resemble clinical populations (based on severity criteria). The remaining papers described interventions for treatment seekers.

Three VRET papers and five papers describing a non-VRET intervention used an active control condition (CBT, Treatment As Usual (TAU), nicotine replacement therapy, imaginal exposure or a form of embodied learning), while seven papers used no control condition, two used a waiting list, one gave access to a self-help manual and one sued a crossover design. Eight papers used a randomized design, yet one study did not compare group differences statistically [21] and one lacked a statistics paragraph in the methods section hindering understanding of their statical approach [58]. Furthermore, only seven studies reported effect sizes. None of the VR2/3 papers described a power analysis, though Malbos et al. [60] refer to a study protocol describing a power analysis. However, they fail to reach the number of participants described in their study protocol [55].

Most papers lacked follow-up data and only report effects directly post-intervention. Those with follow-up data, applied time frames ranging from seven consecutive days following an intervention [48], to one follow-up assessment at 90 days [56] and six months [53], to multiple follow-up assessments during a six-month [58] or 12-months period [49].

In addition to the criteria mentioned in 2.4, several studies only analyzed treatment completers [22,55,58], though drop-out was significant [55,58]. In addition, Malbos [55] specified the total number of completers, not the distribution across treatment and control groups.

**Table 3:** Virtual Reality Exposure Therapy (VRET) studies.

Reference	Design	Population	Control Intervention	VR Intervention	Measurements	Clinical Outcome
**Nicotine studies**						
Lee et al. (2004) [51]	VR2 study with pre-post and per session evaluation	- NTS adolescent males, cigarettes/day = 15.3, mFTQ = 3.6 (*n* = 15)	- No control	- VRET only (*n* = 15) - Computer-generated, multisensory (visual + auditory), complex VE: public bar with proximal cues + agent offering cigarette - No interaction: exposure - 6 × 20 min sessions	Pre-post: - Severity of ND ^a^ Per session: - Cigarettes/morning - Cigarettes/day - Planning of smoking behavior (min) ^b^ - Background craving ^b^ - VR-induced craving ^b^	- n.s. - Reduced (*p* < 0.05) - n.s. - n.s. - n.s. - n.s.
Moon et al. (2009) [52]	VR2 study with per session evaluation	- TS adolescent males, cigarettes/day > 10 (*n* = 8)	- No control	- As in Lee et al. (2004) (*n* = 8) - 6 × 20 min sessions	Per session: - Cigarettes between morning and start VRET - Craving ^c^	- n.s. - n.s.
Pericot-Valverde et al. (2014) [46]	VR2 study with focus on first proof of effectiveness with per session evaluation	- TS, cigarettes/day = 18.2, FTND = 4.8 (*n* = 48)	- No control	- Individualized VRET only (*n* = 48) - Computer-generated, multisensory (visual + auditory), complex VEs with agents: (1) being in a pub, (2) having lunch at home, (3) having breakfast at home, (4) drinking coffee in a cafe, (5) after lunch at restaurant, (6) waiting in the street, (7) watching TV at night, (8) neutral museum - Interaction: with agents + objects via mouse device - 5 weekly, 30 min sessions	Per session: - Cigarettes/day - Air expired CO - Background craving ^d^ - VR-induced craving ^d^	- Reduced (*p* < 0.001, ηp^2^ = 0.82) - Reduced (*p* < 0.001, ηp^2^ = 0.49) - Reduced (*p* < 0.001, ηp^2^ = 0.72) - Reduced (*p* < 0.001, ηp^2^ = 0.66)
Pericot-Valverde et al. (2015) [47]	VR2 study with focus on individual predictors of effectiveness with pre-post session evaluation	- sup>- TS, cigarettes/day = 15.0, FTND = 4.8 (*n* = 41)	- No control	- As in Pericot-Valverde et al. (2014) (*n* = 41) - 5 weekly, 30 min sessions	Pre-post: - VR-induced craving ^d^ Tobacco-dependence related predictors: - Duration of daily smoking - Cigarettes/day - Severity of ND ^e^ - Severity of ND syndrome ^f^ - Severity of nicotine withdrawal ^g^	- Reduced (significance not tested) - n.s. - Increased (*p* = 0.04) - n.s. - n.s. - n.s. - A model with age (*p* = 0.08), marital status (*p* = 0.17), number of cigarettes smoked per day (*p* = 0.04), STAI trait score (*p* = 0.19), BDI-II score (*p* = 0.051) and delay discounting score (*p* = 0.03) explained 25% of variance (*p* = 0.006)
Pericot-Valverde et al. (2016) [48]	VR2 study with focus on application in natural treatment setting with pre-post session evaluation	- TS, cigarettes/day = 17.7, FTND = 4.9 (*n* = 32)	- No control	- Individualized VRET as in Pericot-Valverde et al. (2014) + expired CO feedback + brief advice/counseling (*n* = 32) - 5 weekly, 30 min sessions	Baseline-post first session-post last session: - Background craving ^b^	- Reduced from baseline to post last session (*p* = 0.021, d = 0.44)
Malbos et al. (2018) [55]	VR2/3 study with a clinical population, compared to golden standard (CBT), no follow-up, no power-analysis, use of randomized controlled design	- Ongoing tobacco abstinence (without using NRT or electronic cigarettes) ≥ 7 days, DSM-5 criteria = 5.7–6.3, CDS = 39.1–40.5 (*n* = 61)	- CBT including imaginary exposure (*n* = 31) - 8 weekly, 45 min sessions	- VRET embedded in CBT (*n* = 30) - Computer-generated multisensory (visual, auditory + haptics), contextual/complex VEs with agents: (1) having a drink with people smoking in a virtual beach bar, (2) having dinner with agents smoking on the terrace of a restaurant, (3) being in a furnished living room with an astray and a lighted cigarette, (4) waiting at a bus stop with agents smoking, (5) taking a break in the workplace with colleagues who are smokers, (6) driving a virtual car on a road with colleagues - Interaction: limited (steering wheel) - 8 weekly, 45 min sessions of which 6 sessions contained VRET	Pre-post: - Number of DSM-5 criteria - Severity of ND ^h^ - Background craving ^i^ - Background craving session 3 + 8 ^d^	- n.s. (n^2^ 0.06) - n.s. (n^2^ 0.01) - n.s. (n^2^ 0.02) - n.s. (n^2^ 0.05)
Pericot-Valverde et al. (2019) [49]	VR3 with a clinical population, compared to golden standard (CBT), follow-up, no power-analysis, use of randomized controlled design	- TS, cigarettes/day = 18.7-19.4, FTND = 5.1–5.4 (*n* = 102)	- CBT (*n* = 52) - 6 weekly, 60 min sessions	- CBT + individualized VRET as in Pericot-Valverde et al. (2014), provided immediately before or after the CBT session (*n* = 50) - 6 weekly, 30 min sessions (the first session was used to develop an individual hierarchy for each VE)	Pre-follow up: - VR-induced craving ^d^ - Point-prevalence abstinence - Continuous abstinence	- Reduced across measurements (*p* < 0.001, ηp^2^ = 0.76) and for maximum (*p* < 0.001, ηp^2^ = 0.56) and end scores (*p* < 0.001, ηp^2^ = 0.47) per session - n.s. - Increased relapse between end of treatment and 12 months (*p* = 0.029) in experimental (64.3%) versus control group (37.0%)
Goldenhersch et al. (2020) [56]	VR2 study into adherence and preliminary effectiveness with pre-follow up evaluation and randomized controlled design	- NTS, cigarettes/day = 10.8, FTND =4.5 (*n* = 120)	- Access to a smoking cessation manual (*n* = 60)	- VRET + mindfulness + peer-to-peer support in app + conditional motivational support via SMS or phone call (n = 60) - 360-degree video with a multisensory (visual + auditory) display of proximal VEs that combine (1) the awareness of the act of smoking and (2) the recognition of craving from a perspective of acceptance and commitment - No interaction: exposure - 15 daily, 20 min VRET sessions embedded in broader program	Pre-follow up: - Cigarettes/day Pre-intervention week 1 and 2-postintervention-follow-up - Background craving ^j^ - Severity of ND ^e^ Post: - Treatment retention - Readiness to quit ^k^ - Point-prevalence abstinence Post-follow up: - Sustained abstinence	- Reduced consumption in experimental group at intervention week 3 (*p* = 0.03) and postintervention (*p* < 0.001). Reduced consumption in experimental group between pre and post (*p* < 0.001) pre and follow up (*p* < 0.001) - Reduced craving in experimental group at intervention week 1 and 2 and postintervention (*p* = 0.005) - n.s. within experimental group - 93% in experimental group competed the program. - Increased in experimental group (*p* = 0.005) - Higher postintervention abstinence rate in exp. (23%) versus control group (5%) (*p* = 0.004) - Less relapse in exp. (33%) versus control group (5%) (significance not tested)
**Alcohol studies**						
Hernandez-Serrano et al. (2020) [21]	VR2 study into preliminary effectiveness with pre-post evaluation and randomized controlled design, lacking a direct comparison between groups	- TS outpatients relapsed within 6 months following inpatient TAU, AUDIT = 17.0 (*n* = 42, only completers included)	- TAU (+ 2 VR assessment sessions) (*n* = 27)	- VRET + 2 VRET assessment sessions + TAU (*n* = 15) - Computer-generated, multisensory (visual, auditory + olfactory), complex VEs with agents: (1) bar, (2) restaurant, (3) pub and (4) at home, with a wide variety of alcoholic beverages (bottles of alcohol were displayed in the backgrounds of the VR environments) and different times of the day (daytime or night-time). The olfactory stimulus was provided by transferring a small amount of an alcoholic beverage, corresponding to the alcoholic drinks being displayed in the VE, onto cotton pads and placed close to each participant - Interaction: full-body interaction (incl. grabbing with Oculus touch controllers) - 6 × 50 min twice/weekly sessions	Pre-post: - Craving ^l^	- Reduced in VR-CET +TAU group (*p* = 0.003), but n.s. in TAU
**Gambling studies**						
Giroux et al. (2013) [54]	VR2 study into preliminary effectiveness with pre-post evaluation	- NTS gamblers, CPGI = 9.9 (*n* = 10)	- No control	- VRET only (*n* = 10) - Computer-generated, contextual and complex, multisensory (visual + auditory) cues: facing bank machine, bar counter, looking at the VLTs and gamblers and select a free VLT and sit down to play without playing. This action sequence was repeated five times. - Interaction: moving via mouse device - 1 × 20 min session	Pre-post: - Urge to gamble ^b^ - Perceived self-efficacy ^b^	- n.s. - n.s.

Abbreviations: AUDIT = Alcohol Use Disorder Identification Test; CBT = Cognitive-Behavioral Therapy; CDS = Cigarette Dependence Scale; CPGI = Canadian Problem Gambling Index; DSM-5 = Diagnostic and Statistical Manual of Mental Disorders, 5th edition; FTND = Fagerstrom Test of Nicotine Dependence; HSD = Heavy social drinkers; LD = Light Drinkers; MD = Methamphetamine Dependent (according to DSM-IV criteria (Diagnostic and Statistical Manual of Mental Disorders, 4th edition)); mFTQ = modified Fagerstrom Tolerance Questionnaire; ND = Nicotine Dependent; NTS = Non-Treatment Seeking; RCT = Randomized Clinical Trial; TAU = Treatment As Usual; TS = Treatment Seeking; VEs = Virtual Environments; VLT = Video Lottery Terminal; VRET = Virtual Reality Exposure Therapy. Measurement instruments: ^a^ modified Fagerstrom Tolerance Questionnaire (mFTQ); ^b^ Likert-type scale(s); ^c^ Unspecified; ^d^ Visual Analogue Scale (VAS); ^e^ Fagerstrom Test of Nicotine Dependence (FTND); ^f^ Nicotine Dependence Syndrome Scale (NDSS); ^g^ Minnesota Nicotine Withdrawal Scale (MNWS); ^h^ Cigarette Dependence Scale (CDS); ^i^ Tobacco Craving Questionnaire (TCQ); ^j^ Questionnaire of smoking Urges (QSU); ^k^ Contemplation Ladder; ^l^ Multidimensional Alcohol Craving Scale—Virtual Reality (MACS-VR).

**Table 4:** Other Virtual Reality (VR) treatment studies.

Reference	Design	Population	Control Intervention	VR Intervention	Measurements	Clinical Outcome
**Nicotine studies**						
Girard et al. (2009) [53]	VR2/3 study into preliminary effectiveness with a clinical population, not compared to golden standard, follow-up, no power-analysis, use of randomized controlled design	- NTS, FTND = 5.9–6.4 (*n* = 91)	- VR-embodied learning: find and grasp up to 60 virtual balls, embedded in broader psychosocial program (*n* = 45)	- VR-embodied learning, embedded in broader psychosocial program (*n* = 46) - Computer-generated, multisensory (visual + auditory), proximal VEs: (1) medieval castle/find and crush cigarettes - Interaction: Gamepad to control virtual arm - 4 weekly, 30 min VR sessions in first 4 weeks of psychosocial program (whole program consisted of 8 sessions in week 1, 2, 3, 4, 6, 8, 10 and 12)	Pre-post-follow up: - Severity of ND ^a^ Post VR-post program: - Cigarettes/day + air expired CO Follow up: - Cigarettes/day	- Stronger reduction in experimental group (*p* < 0.05), most notably from week 4 onwards (*p* < 0.001) - Abstinence status: post VR 2% (experimental group) versus 9% (control group) (n.s.), post program increased to 15% (experimental group) versus 2% (control group) (*p* < 0.05) - Abstinence status (past week) 39% (experimental group) versus 20% (control group) (*p* < 0.05)
Bordnick et al. (2012) [58]	VR2 study into feasibility and preliminary effectiveness with pre-follow up and post-session evaluation and randomized controlled design	- TS, cigarettes/day = 24.5–26.4, FTND = 5.9–6.6 (*n* = 46, only completers included)	- NRT-only (*n* = 25)	- Progressive individualized exposure + coping skill training + NRT (*n* = 21) - Computer-generated, multisensory (visual, auditory, olfactory + haptics), complex VE: (1) party, (2) driving, (3) restaurant, (4) office building and courtyard, (5) convenience store, (6) airport smoking lounge and gate - Interaction: no/limited - 10 weekly, 60 min sessions	Post (between-groups): - Cigarettes/week - Craving ^b^ - Self-efficacy ^c^ Pre-follow up: - Cigarettes/day - Confidence to resist smoking ^d^	- Reduced (*p* < 0.05, ηp^2^ = 0.14) - Reduced (*p* < 0.05, ηp^2^ = 0.37) - Increased (*p* < 0.05, ηp^2^ = 0.13) - Reduced at 1, 2 (*p* < 0.05) + 6 months (*p* < 0.01) follow up - Increased at 1, (*p* < 0.05) 2, 3 (*p* < 0.01) and 6 (*p* < 0.001) months follow up
Caponnetto et al. (2018) [59]	VR2 study into feasibility and preliminary effectiveness with pre-post session evaluation	- NTS, cigarettes/day = 15, FTND = 5.3, not motivated to quit (*n* = 40)	- No control	- VR-covert sensitization (*n* = 40) - Shocking image, video and VR session are compared in randomized cross-over design - Computer-generated, multisensory (visual + auditory), proximal VE that changes over time from neutral to aversive: cigarettes and smoke - Interaction: full body (magic leap) - 3 × 15–30 min, 2 days in between sessions	Pre-post: - Motivation to quit ^e^	- Increase: image < video < VR (*p* < 0.01, η^2^ = 0.95)
**Alcohol studies**						
Spagnoli et al. (2014) [57]	VR2 study into preliminary effectiveness with pre-post session evaluation	- TS alcohol drinkers (*n* = 50)	- Traditional assessment only (*n* = 25)	- VR + traditional assessment (*n* = 25) - Computer-generated, complex VEs (one neutral, two involving alcohol cues and one a performance task) - Interaction: interaction with gamepad - 1 session	Pre-post: - Readiness to change ^f^ Precontemplation phase Contemplation phase Determination phase Termination phase Action phase Maintenance phase - Self-efficacy ^g^	- n.s. - n.s. (*p* = 0.052) - n.s. - Increased (*p* = 0.009) - n.s. - n.s. - Increased (*p* = 0.002)
Choi & Lee (2015) [50]	VR2 study with focus on preliminary effectiveness with pre-post session evaluation, within-person controlled	- sup>- NTS male under-graduates, AUDIT HSD = 20.0, LD = 4.6 (*n* = 40)	- sup>- No control (HSD (*n* = 20) and LD (*n* = 20) are compared in cross-over design)	- VR-covert sensitization (*n* = 40) - Computer-generated, aversive, multisensory (visual + auditory), context environments: (1) virtual hospital, (2) virtual subway - Interaction: Keypad - 1 × 20 min session	Pre-post: - Craving ^h^ - Implicit alcohol associations ^i^ - Implicit alcohol eye behavior ^j^ - Implicit alcohol attentional bias ^k^	- HSD showed a greater reduction than LD group (*p* < 0.01) - HSD showed a weaker positive association than LD group (*p* < 0.01) - Reduced dwell time in both HSD and LD group (*p* < 0.05, η^2^ = 0.14) - Reduced reaction times in both HSD and LD group (*p* < 0.05, η^2^ = 0.14)
**Methamphetamine studies**						
Wang et al. (2019) [22]	VR2 studies with focus on preliminary effectiveness with pre-post session evaluation and randomized controlled designs	- Study 1: TS males with MD (*n* = 61) - Study 2: abstaining methamphetamine abusers (*n* = 888, only completers included)	- Waiting list (*n* = 30) - Waiting list (*n* = 276)	- Study 1 (*n* = 31) + 2 (*n* = 612): - VR-covert sensitization - 360°, aversive, multisensory (visual + auditory) complex VEs. Scene1: auditory cues with social interaction; Scene 2: drugs and drug-related paraphernalia; Scene 3: METH-use social context). In the second part of the videos, participants viewed that characters in the videos experienced a distinct adverse consequence caused by METH use, totaling six videos. - No interaction: exposure - 5 × 6 min sessions, twice weekly	Study 1, pre-post: - Craving ^e^ - Liking ^e^ - Propensity to use ^e^ Study 2, pre-post: - ECG (HRV indexes)	- Reduced (*p* = 0.001) - Reduced (*p* = 0.002) - n.s. - SDNN, RMSSD, pNN50 reduced (*p* < 0.001), nLF, nHF, LF/HF n.s.
**Gambling studies**						
Bouchard et al. (2017) [45]	VR2 studies with focus on integrating VR and CBT (study 2) and preliminary effectiveness (study 3) with pre-post session evaluation and randomized controlled design	- Study 2: TS inpatient pathological gamblers, SOGS = 11.5 (*n* = 34) - Study 3: TS pathological gamblers, CPGI = 20.0 (*n* = 25)	- 2 imaginal exposure sessions embedded in 28-day CBT program (*n* = 14) - Imaginal exposure (4 sessions) (*n* = 11)	- VR-CBT: a hierarchy in exposure to a virtual bar or casino guides users progressively approaching machines where they can gamble. Users are invited to walk to each step of the hierarchy and apply various CBT techniques. The VR-CBT was embedded in 28-day CBT program. - Computer-generated, multisensory (visual, auditory, haptics), complex VEs: (1) generated virtual casino, (2) generated virtual bar, with fixed locations in the VEs to explore and the sound controlled by proximity or therapist. - Interaction: with agents and objects via mouse device (wireless mouse) and a box with pushbuttons replicating the interface panel of a real VLT. - Study 2: 2 VR-CBT sessions - (*n* = 20) - Study 3: 4 VR-CBT sessions - (*n* = 14)	Study 2, pre-post: - Craving ^l^ Study 3, pre-post: - Severity of problem gambling ^m^ - Number of diagnostic criteria ^o^ - Gambling related cognitions ^p^	- Time *p* < 0.001, group + interaction n.s. (η^2^ = 0.006) - n.s. (ηp^2^ = 0.001) - n.s. (ηp^2^ = 0.07) - n.s. (ηp^2^ = 0.04)

Abbreviations: AUDIT = Alcohol Use Disorder Identification Test; CBT = Cognitive-Behavioral Therapy; CG = control group; CO = carbon monoxide; CPGI = Canadian Problem Gambling Index; FTND = Fagerstrom Test of Nicotine Dependence; IG = intervention group; MD = Methamphetamine Dependent (according to DSM-IV criteria (Diagnostic and Statistical Manual of Mental Disorders, 4th edition)); ND = Nicotine Dependent; NRT = Nicotine Replacement Therapy; NTS = Non-Treatment Seeking; RCT = Randomized Clinical Trial; TS = Treatment Seeking; VEs = virtual environments; VLT = Video Lottery Terminal; Measurement instruments: ^a^ Fagerstrom Test of Nicotine Dependence (FTND); ^b^ Questionnaire of smoking Urges-Brief version (QSU-brief); ^c^ Smoking Abstinence Self-Efficacy (SASE); ^d^ modified Smoking Confidence Questionnaire (mSCQ); ^e^ Visual Analogue Scale (VAS); ^f^ Motivation Assessment of Change questionnaire-Alcoholism version (MAC2-A); ^g^ Generalized Self- Efficacy Questionnaire (GSE); ^h^ Alcohol Urge Questionnaire (AUQ); ^i^ Alcohol-Implicit Association Task (IAT); ^j^ Eye-tracking test; ^k^ Alcohol-Stroop test; ^l^ Gambling Craving Scale (GCS); ^m^ Canadian Problem Gambling Index (CPGI); ^o^ Diagnostic Interview for Gambling (DIG); ^p^ Gambling Related Cognition Scale (GRCS).

## 4. Discussion

### 4.1. General Discussion

The present systematic review evaluated (1) the diagnostic/prognostic value of VR-induced cue-reactivity for the clinical assessment of patients with ADs and (2) the effectiveness of VR-delivered treatment in patients with ADs. Though the number of papers on application of VR in ADs has grown over the past decade, study methods and outcome measures, and consequently results, were highly heterogeneous. In addition, most studies lack a clinical focus to demonstrate the (added) value in clinical practice.

Regarding VR-assessment, our findings show that cue-reactivity paradigms might be of diagnostic value in patients with nicotine, alcohol and methamphetamine use disorder, as well as gaming disorder. Despite negative findings in some studies, the majority (*n* = 15/19) reported one or more significant associations between clinical status (dependence status, dependence severity) and VR-cue-reactivity (craving, psychophysiology and withdrawal).

Regarding VR-treatment, one VRET trial showed a negative effect compared to standard CBT treatment in tobacco use disorder [49], with other VRET pilot studies not showing convincing treatment effects either [21,46,47,48,51,52,54,55,56]. Similarly, a gambling study using VRET did not show significant effects of a single session on the urge to gamble or self-efficacy [54]. Likewise, series of pilot studies using VR-CBT did not show significant added value compared to TAU either [45]. Other VR interventions, such as embodied learning (crushing cigarettes) [53], coping skills training (nicotine) [58] and aversive learning (methamphetamine) [22,50,59] produced encouraging results, with beneficial effects on disease severity and abstinence rates.

Our findings show that clinical assessment studies toward the diagnostic and prognostic potential of VR-induced cue-reactivity are scarce. Previous reviews showed that VR can induce craving in different VEs, with various cue exposure procedures [12,13,14,15,16], but lack insights into the clinical value [18]. We extend this body of evidence by exploring the diagnostic value of VR and reviewing studies that relate cue-reactivity to clinical indices. However, only two discriminative studies were identified, comparing AD patients with healthy controls, using psychophysiological measures during VR-cue-exposure [23,28]. In addition, several studies showed a relationship between VR-cue-reactivity and the severity of various clinical parameters. Given the limited number of discriminative studies and heterogeneity of methods and results, it remains to be elucidated whether VR-assessment can add to current clinical assessment practice. Further research into both discrimination between healthy and AD populations and severity assessment within AD populations is warranted.

Interestingly, while craving has been considered a predictive factor for treatment success or relapse [61,62,63], we did not identify prognostic studies that investigated the association between reactivity to VR-cue-exposure and treatment outcome. Only one study showed an effect of pharmacological treatment (nicotine lozenge) on VR-cue-reactivity [36]. Furthermore, previous studies using a VR-based assessment in patients with AUD reported greater exhaustion compared to standard clinical interviews [57,64]. Therefore, future studies should assess the feasibility and acceptability of VR assessments, as well as its predictive value in clinical practice [12], and explore potential benefits of VR assessment as compared to non-VR induced cue-reactivity, with more easy-to-use and more comprehensive procedures, such as personalized environments or smartphone applications.

Regarding VRET, the clinical value in ADs remains unclear. We only identified a single clinical effect study (level VR3), showing negative effects of the VR intervention [49] and several pilot studies (level VR2) showing limited effectiveness [21,46,47,48,51,52,54,55,56]. This is in line with previous reviews on the efficacy of traditional ET in ADs, also showing no to small effects or even negative effects [65,66]. Several studies in this review showed short-term reductions in cue-reactivity but did not examine long-term effects on extinction (i.e., spontaneous recovery, reinstatement, renewal effect) due to missing follow-ups [66]. It could be argued that long-term outcome might even be worse due to potential reinstatement effects of ET, as observed in studies on face-to-face ET [67]. Considering the limited short-term effects of VRET, it could also be argued, that ecological validity might not improve ET efficacy as expected [12,15,16]. Yet, recent advancements in VR technology, such as display technology, multimodality and multi-sensuality, might increase the ecological validity of VRET and need further study in the treatment of patients with ADs [15].

Other VR-based treatment approaches, such as VR-CBT [45], -embodied learning [53], -covert sensitization [22,50,59] and -cognitive reframing [57], showed somewhat more promising results, though the level of evidence is still limited (mainly pilot studies at level VR2). These findings are in line with previous reviews that suggest the incorporation of CBT-related coping skills training in VR-based treatments to transfer VR-based learning effects to everyday experience [12,15,16]. Using a VR in which the patient is an actor rather than passive observer may be more beneficial [53,58]. As described by Segawa et al. [12], embodied experiences could empower the patient’s self-regulation and self-efficacy to foster sustainable behavioral change and improve coping with cue-reactivity [12,68]. Future studies should disentangle the most effective VR components and procedures to maximize such learning experiences. Likewise, the added value of VR treatment on top of traditional approaches or as an alternative to face-to-face sessions needs to be examined [69].

Throughout the review process, we encountered fundamental methodological shortcomings in many of the identified papers. As mentioned before, VR-assessment techniques, study procedures and instruments highly varied, limiting the comparability between studies and development of best practices for future research. The included studies were mostly in developmental stages and were not set up to examine diagnostic possibilities for the clinical application. To further investigate the diagnostic capabilities, the sensitivity, specificity and predictive values should be examined in random samples and compared to golden standards. Thus, representative clinical populations need to be studied to avoid an over- or underestimation of predictive values.

Treatment studies were mostly piloting stages (level VR2) and lacked detailed intervention protocols, predefined primary outcome measures, control conditions, randomization, follow-up data and sufficient sample sizes based on power analyses. Multiple studies failed to clearly communicate the conducted procedures and technological details, resulting in low explanatory power and the inability to replicate findings [16,50]. Besides, protocols delivered in VR were often vaguely defined [12,45,53,58,59]; for instance, one is described as VR-counterconditioning while VR-covert sensitization was applied [22]. Furthermore, VRET approaches must expose participants to a variety of VEs until craving is reduced, without acting upon the craving elicited, otherwise the participants may be sensitized instead. Moreover, time to extinction or a certain level of reduction in craving needs to be examined, to be sure of extinction effects, instead of standard exposure times regardless of the participant’s response. Hence, VR research in ADs should focus on method development and reporting with scientific rigor, including evidence-based protocols and clear clinical endpoints and pre-registration of clinical trials.

A topic that has largely been overlooked in the VR addiction field is the ethics of VR application in a vulnerable population with mental health problems [12,70,71]. Kellmeyer et al. describe two main issues related to the development of VR applications in psychiatric patients [70]. The deceptive illusion and persuasiveness of the VEs might influence the user’s behavior and ability to differentiate reality from virtuality. Side-effects of VR therapy, such as cybersickness and discomfort after prolonged use, should be considered thoroughly, but were not reported in the included studies [72]. Likewise, aversive conditioning remains ethically controversial and might be prone to cultural influences. Lastly, data-collecting HMDs threaten the patient’s privacy, and might restrict the implementation and benefits of cutting-edge VR hardware in clinical practice [71]. Therefore, ethical guidelines should be established that addressed the aforementioned issues, for example through a patient-centered design and value alignment when developing future VR systems [70].

### 4.2. Strengths and Limitations

In comparison to previous review papers, first and foremost we focused on clinical applicability of the findings, differentiating between promising new concepts and evidence that can be applied in the clinical context. We were able to identify several additional papers that were either published in the past years [20,21,22,23,35,36,42,43,56] or through an additional search in the PsycINFO database [32,41,48,55]. In addition, we limited our review to studies applying HMDs, excluding other devices which are not state of the art and more difficult to apply in clinical practice. There are, however, several limitations that should be considered when interpreting the results of this systematic review. We were unable to systematically assess the quality of the studies through standard clinical frameworks due to the early stage of intervention development and lack of methodological details described in many studies [26]. Other limitations in the field include small sample sizes, heterogeneous methodologies and group characteristics, as well as a lack of validated instruments used to measure clinically relevant outcomes, accompanied by a lack of adequate follow-up periods and control groups. Therefore, a systematic quality appraisal with standardized clinical frameworks or meta-analysis of data was not possible [73,74]. Another issue is that we excluded two papers from the review because it was unclear whether HMDs were used, because of a lack of detailed methodological reporting [75,76]. These methodological shortcomings need to be addressed to further the VR field and bring VR technology to clinical care for patients with ADs.

### 4.3. Future Outlook

Future research needs to circumvent the current methodological shortcomings through scientific rigor with clear, pre-registered clinical endpoints. Assessment studies should investigate the potential of cue-reactivity to diagnose ADs (sensitivity and specificity), discriminate different levels of AD severity, monitor treatment effects and predict treatment outcome or relapse. The combination of multiple parameters into a discriminative model, for instance through machine learning, seems promising. The resulting models need to be tested in representative clinical populations to avoid biased conclusions, and should be compared to regular diagnostic instruments (e.g., DSM5, ICD10) as well as alternative, less complex approaches.

Treatment studies should focus on the implementation of therapeutic elements in the VE design (e.g., coping skills training, mindfulness) and the related development of treatment protocols that entail active (embodied) learning practices. To evaluate the (cost-)effectiveness, relevant RCTs (level VR3), including adequate follow-up periods, need to be conducted. During our literature review, we identified five study protocols that report on planned RCTs and insights into potential new treatment mechanisms [77,78,79,80,81]. The studies focus on approach-avoidance training, mindfulness-based relapse prevention, memory-retrieval extinction and the use of pharmacotherapy (Isradipine) to enhance the effect of VRET on the extinction of craving. However, the interdependence of psychological mechanisms and the technological implementation thereof should receive more attention to foster the identification of effective VR treatment paradigms.

## 5. Conclusions

The studies on VR in addiction medicine show that benefits for the clinical practice remain to be elucidated. Though we found 19 papers reporting a relation between cue-reactivity and one or more clinical parameters, thereby providing some insight in the potential diagnostic value of VR-induced cue-reactivity, only two studies specifically analyzed the discriminative power of the VR intervention, and are therefore considered to be clinical assessment studies. Regarding VR-treatment, VRET studies showed conflicting results. While the application of VR-CBT, -embodied learning and -covert sensitization shows promising paradigms, which up to now lack clinical effect studies. Thus, VR in ADs is not yet an intervention that is ready for clinical application beyond clinical studies. A major issue in this field of research is a general lack of methodological rigor and insufficient quality of reporting methods. To move the field forward, studies with clear clinical endpoints and scientific quality, including randomized controlled designs and adequate follow-up, are required.

## Figures and Tables

**Table 1:** PICOS framework to identify eligibility criteria.

Population:	Adolescent or adult humans with addictive disorders (SUD or other addictive behaviors) or daily/heavy use
Intervention *:	Immersive VR (using Head-Mounted Display) for the assessment or treatment of addictive disorders
Comparators *:	No limitation
Outcomes:	Assessment: Diagnosis, disease severity, measure of treatment effect, or predictor of treatment outcome, related to VR-cue-reactivity (e.g., craving, psychophysio-logical response and attention to cues) Treatment: Cue-reactivity, motivation, dependence severity, substance use, abstinence
Study designs:	No limitation, except single case studies (*n* < 3)
Timing:	No restriction
Language:	English

* Does not apply to the (1) research question focusing on assessment.

## Data Availability

The datasets generated for this study are available on request to the corresponding author.

## Supplementary Materials

The following are available online at https://www.mdpi.com/article/10.3390/jcm10163658/s1, Table S1: Definitions—VR therapy in Ads.

## References

[1] Rehm J., Shield K.D. (2019). Global burden of disease and the impact of mental and addictive disorders. Curr. Psychiatry Rep..

[2] Gowing L.R., Ali R.L., Allsop S., Marsden J., Turf E.E., West R., Witton J. (2015). Global statistics on addictive behaviours: 2014 status report. Addiction.

[3] Schellekens A., De Jong C., Buitelaar J., Verkes R. (2015). Co-morbid anxiety disorders predict early relapse after inpatient alcohol treatment. Eur. Psychiatry.

[4] Lappan S.N., Brown A.W., Hendricks P.S. (2020). Dropout rates of in-person psychosocial substance use disorder treatments: A systematic review and meta-analysis. Addiction.

[5] Ferreri F., Bourla A., Mouchabac S., Karila L. (2018). e-Addictology: An overview of new technologies for assessing and intervening in addictive behaviors. Front. Psychiatry.

[6] Emmelkamp P.M., Meyerbröker K. (2021). Virtual Reality Therapy in Mental Health. Annu. Rev. Clin. Psychol..

[7] Bown J., White E., Boopalan A. (2017). Looking for the ultimate display: A brief history of virtual reality. Boundaries of Self and Reality Online.

[8] Freeman D., Reeve S., Robinson A., Ehlers A., Clark D., Spanlang B., Slater M. (2017). Virtual reality in the assessment, understanding and treatment of mental health disorders. Psychol. Med..

[9] Oing T., Prescott J. (2018). Implementations of virtual reality for anxiety-related disorders: Systematic review. JMIR Serious Games.

[10] Carl E., Stein A.T., Levihn-Coon A., Pogue J.R., Rothbaum B., Emmelkamp P., Asmundson G.J., Carlbring P., Powers M.B. (2019). Virtual reality exposure therapy for anxiety and related disorders: A meta-analysis of randomized controlled trials. J. Anxiety Disord..

[11] Rus-Calafell M., Garety P., Sason E., Craig T.J., Valmaggia L.R. (2018). Virtual reality in the assessment and treatment of psychosis: A systematic review of its utility, acceptability and effectiveness. Psychol. Med..

[12] Segawa T., Baudry T., Bourla A., Blanc J.-V., Peretti C.-S., Mouchabac S., Ferreri F. (2020). Virtual reality (VR) in assessment and treatment of addictive disorders: A systematic review. Front. Neurosci..

[13] Pericot-Valverde I., Germeroth L.J., Tiffany S.T. (2016). The use of virtual reality in the production of cue-specific craving for cigarettes: A meta-analysis. Nicotine Tob. Res..

[14] Hone-Blanchet A., Wensing T., Fecteau S. (2014). The use of virtual reality in craving assessment and cue-exposure therapy in substance use disorders. Front. Hum. Neurosci..

[15] Ghiţă A., Gutiérrez-Maldonado J. (2018). Applications of virtual reality in individuals with alcohol misuse: A systematic review. Addict. Behav..

[16] Trahan M.H., Maynard B.R., Smith K.S., Farina A.S., Khoo Y.M. (2019). Virtual reality exposure therapy on alcohol and nicotine: A systematic review. Res. Soc. Work Pract..

[17] Bell I.H., Nicholas J., Alvarez-Jimenez M., Thompson A., Valmaggia L. (2020). Virtual reality as a clinical tool in mental health research and practice. Dialogues Clin. Neurosci..

[18] Carter B.L., Tiffany S.T. (1999). Meta-analysis of cue-reactivity in addiction research. Addiction.

[19] American Psychiatric Association (2013). Diagnostic and Statistical Manual of Mental Disorders (DSM-5^®^).

[20] Ghiţă A., Hernández-Serrano O., Fernández-Ruiz Y., Monras M., Ortega L., Mondon S., Teixidor L., Gual A., Porras-García B., Ferrer-García M. (2019). Cue-Elicited Anxiety and Alcohol Craving as Indicators of the Validity of ALCO-VR Software: A Virtual Reality Study. J. Clin. Med..

[21] Hernández-Serrano O., Ghiţă A., Figueras-Puigderrajols N., Fernández-Ruiz J., Monras M., Ortega L., Mondon S., Teixidor L., Gual A., Ugas-Ballester L. (2020). Predictors of Changes in Alcohol Craving Levels during a Virtual Reality Cue Exposure Treatment among Patients with Alcohol Use Disorder. J. Clin. Med..

[22] Wang Y.-G., Liu M.-H., Shen Z.-H. (2019). A virtual reality counterconditioning procedure to reduce methamphetamine cue-induced craving. J. Psychiatr. Res..

[23] Ding X., Li Y., Li D., Li L., Liu X. (2020). Using machine-learning approach to distinguish patients with methamphetamine dependence from healthy subjects in a virtual reality environment. Brain Behav..

[24] Moher D., Liberati A., Tetzlaff J., Altman D.G., Group P. (2009). Preferred reporting items for systematic reviews and meta-analyses: The PRISMA statement. PLoS Med..

[25] Schardt C., Adams M.B., Owens T., Keitz S., Fontelo P. (2007). Utilization of the PICO framework to improve searching PubMed for clinical questions. BMC Med. Inform. Decis. Mak..

[26] Birckhead B., Khalil C., Liu X., Conovitz S., Rizzo A., Danovitch I., Bullock K., Spiegel B. (2019). Recommendations for methodology of virtual reality clinical trials in health care by an international working group: Iterative study. JMIR Ment. Health.

[27] Higgins J.P., Thomas J., Chandler J., Cumpston M., Li T., Page M.J., Welch V.A. (2019). Cochrane Handbook for Systematic Reviews of Interventions.

[28] Wang Y.-G., Shen Z.-H., Wu X.-C. (2018). Detection of patients with methamphetamine dependence with cue-elicited heart rate variability in a virtual social environment. Psychiatry Res..

[29] Ferrer-Garcia M., Garcia-Rodriguez O., Gutiérrez-Maldonado J., Pericot-Valverde I., Secades-Villa R. (2010). Efficacy of virtual reality in triggering the craving to smoke: Its relation to level of presence and nicotine dependence. Stud. Health Technol. Inf..

[30] Ferrer-García M., García-Rodríguez O., Pericot-Valverde I., Yoon J.H., Secades-Villa R., Gutiérrez-Maldonado J. (2012). Predictors of smoking craving during virtual reality exposure. Presence Teleoperators Virtual Environ..

[31] García-Rodríguez O., Pericot-Valverde I., Gutiérrez-Maldonado J., Ferrer-García M., Secades-Villa R. (2012). Validation of smoking-related virtual environments for cue exposure therapy. Addict. Behav..

[32] Bordnick P.S., Yoon J.H., Kaganoff E., Carter B. (2013). Virtual reality cue reactivity assessment: A comparison of treatment-vs. nontreatment-seeking smokers. Res. Soc. Work Pract..

[33] Pericot-Valverde I., García-Rodríguez O., Rus-Calafell M., Fernández-Artamendi S., Ferrer-García M., Gutiérrez-Maldonado J. (2013). Peak provoked craving after smoking cessation. Annu. Rev. Cyberther. Telemed..

[34] Thompson-Lake D.G., Cooper K.N., Mahoney III J.J., Bordnick P.S., Salas R., Kosten T.R., Dani J.A., De La Garza R. (2015). Withdrawal symptoms and nicotine dependence severity predict virtual reality craving in cigarette-deprived smokers. Nicotine Tob. Res..

[35] de Bruijn G.-J., de Vries J., Bolman C., Wiers R. (2021). (No) escape from reality? Cigarette craving in virtual smoking environments. J. Behav. Med..

[36] Kotlyar M., Vogel R.I., Dufresne S.R., Mills A.M., Vuchetich J.P. (2020). Effect of nicotine lozenge use prior to smoking cue presentation on craving and withdrawal symptom severity. Drug Alcohol Depend..

[37] Bordnick P.S., Traylor A., Copp H.L., Graap K.M., Carter B., Ferrer M., Walton A.P. (2008). Assessing reactivity to virtual reality alcohol based cues. Addict. Behav..

[38] Lee J.S., Namkoong K., Ku J., Cho S., Park J.Y., Choi Y.K., Kim J.-J., Kim I.Y., Kim S.I., Jung Y.-C. (2008). Social pressure-induced craving in patients with alcohol dependence: Application of virtual reality to coping skill training. Psychiatry Investig..

[39] Ryan J.J., Kreiner D.S., Chapman M.D., Stark-Wroblewski K. (2010). Virtual reality cues for binge drinking in college students. CyberPsychol. Behav. Soc. Netw..

[40] Traylor A.C., Parrish D.E., Copp H.L., Bordnick P.S. (2011). Using virtual reality to investigate complex and contextual cue reactivity in nicotine dependent problem drinkers. Addict. Behav..

[41] Ghiţă A., Ferrer-Garcia M., Gutiérrez-Maldonado J. (2017). Behavioral, craving and anxiety responses among light and heavy drinking college students in alcohol-related virtual environments. Annu. Rev. Cyberther. Telemed.

[42] Simon J., Etienne A.-M., Bouchard S., Quertemont E. (2020). Alcohol craving in heavy and occasional alcohol drinkers after cue exposure in a virtual environment: The role of the sense of presence. Front. Hum. Neurosci..

[43] Tan H., Chen T., Du J., Li R., Jiang H., Deng C.-l., Song W., Xu D., Zhao M. (2019). Drug-related Virtual Reality Cue Reactivity is Associated with Gamma Activity in Reward and Executive Control Circuit in Methamphetamine Use Disorders. Arch. Med. Res..

[44] Shin Y.-B., Kim J.-J., Kim M.-K., Kyeong S., Jung Y.H., Eom H., Kim E. (2018). Development of an effective virtual environment in eliciting craving in adolescents and young adults with internet gaming disorder. PLoS ONE.

[45] Bouchard S., Robillard G., Giroux I., Jacques C., Loranger C., St-Pierre M., Chrétien M., Goulet A. (2017). Using virtual reality in the treatment of gambling disorder: The development of a new tool for cognitive behavior therapy. Front. Psychiatry.

[46] Pericot-Valverde I., Secades-Villa R., Gutiérrez-Maldonado J., García-Rodríguez O. (2014). Effects of systematic cue exposure through virtual reality on cigarette craving. Nicotine Tob. Res..

[47] Pericot-Valverde I., García-Rodríguez O., Gutiérrez-Maldonado J., Secades-Villa R. (2015). Individual variables related to craving reduction in cue exposure treatment. Addict. Behav..

[48] Pericot-Valverde I., Ferrer-Garcia M., Pla-Sanjuanelo J., Secades-Villa R., Gutiérrez J. (2016). Cue exposure treatment through virtual reality reduce cigarette craving in real life environments. Annu. Rev. Cyberther. Telemed..

[49] Pericot-Valverde I., Secades-Villa R., Gutiérrez-Maldonado J. (2019). A randomized clinical trial of cue exposure treatment through virtual reality for smoking cessation. J. Subst. Abus. Treat..

[50] Choi Y.J., Lee J.-H. (2015). The effect of virtual covert sensitization on reducing alcohol craving in heavy social drinkers. Virtual Real..

[51] Lee J., Lim Y., Graham S.J., Kim G., Wiederhold B.K., Wiederhold M.D., Kim I.Y., Kim S.I. (2004). Nicotine craving and cue exposure therapy by using virtual environments. CyberPsychol. Behav..

[52] Moon J., Lee J.-H. (2009). Cue exposure treatment in a virtual environment to reduce nicotine craving: A functional MRI study. CyberPsychol. Behav..

[53] Girard B., Turcotte V., Bouchard S., Girard B. (2009). Crushing virtual cigarettes reduces tobacco addiction and treatment discontinuation. CyberPsychol. Behav..

[54] Giroux I., Faucher-Gravel A., St-Hilaire A., Boudreault C., Jacques C., Bouchard S. (2013). Gambling exposure in virtual reality and modification of urge to gamble. Cyberpsychol. Behav. Soc. Netw..

[55] Malbos E., Borwell B., Cantalupi R., Lancon C. (2018). Virtual Reality Cue Exposure for Smoking Relapse Prevention: A Comparative Trial. Annu. Rev. Cybertherapy Telemed..

[56] Goldenhersch E., Thrul J., Ungaretti J., Rosencovich N., Waitman C., Ceberio M.R. (2020). Virtual Reality Smartphone-Based Intervention for Smoking Cessation: Pilot Randomized Controlled Trial on Initial Clinical Efficacy and Adherence. J. Med. Internet Res..

[57] Spagnoli G., Gatti E., Massari R., Sacchelli C., Riva G. (2014). Can virtual reality be useful to assess subjects with alcohol dependency? Development of a new assessment protocol for patients with alcoholism. Eur. Int. J. Sci. Technol..

[58] Bordnick P.S., Traylor A.C., Carter B.L., Graap K.M. (2012). A feasibility study of virtual reality-based coping skills training for nicotine dependence. Res. Soc. Work Pract..

[59] Caponnetto P., Maglia M., Lombardo D., Demma S., Polosa R. (2018). The role of virtual reality intervention on young adult smokers’ motivation to quit smoking: A feasibility and pilot study. J. Addict. Dis..

[60] Giovancarli C., Malbos E., Baumstarck K., Parola N., Pélissier M.-F., Lançon C., Auquier P., Boyer L. (2016). Virtual reality cue exposure for the relapse prevention of tobacco consumption: A study protocol for a randomized controlled trial. Trials.

[61] Paliwal P., Hyman S.M., Sinha R. (2008). Craving predicts time to cocaine relapse: Further validation of the Now and Brief versions of the cocaine craving questionnaire. Drug Alcohol Depend..

[62] Galloway G.P., Singleton E.G., Methamphetamine Treatment Project Corporate Authors (2008). How long does craving predict use of methamphetamine? Assessment of use one to seven weeks after the assessment of craving. Subst. Abus. Res. Treat..

[63] Perkins K.A. (2011). Subjective reactivity to smoking cues as a predictor of quitting success. Nicotine Tob. Res..

[64] Gatti E., Massari R., Sacchelli C., Lops T., Gatti R., Riva G. (2008). Why do you drink? Virtual reality as an experiential medium for the assessment of alcohol-dependent individuals. Stud. Health Technol. Inform..

[65] Mellentin A.I., Skøt L., Nielsen B., Schippers G.M., Nielsen A.S., Stenager E., Juhl C. (2017). Cue exposure therapy for the treatment of alcohol use disorders: A meta-analytic review. Clin. Psychol. Rev..

[66] Conklin C.A., Tiffany S.T. (2002). Applying extinction research and theory to cue-exposure addiction treatments. Addiction.

[67] Marissen M.A., Franken I.H., Blanken P., van den Brink W., Hendriks V.M. (2007). Cue exposure therapy for the treatment of opiate addiction: Results of a randomized controlled clinical trial. Psychother. Psychosom..

[68] Riva G., Serino S., Di Lernia D., Pavone E.F., Dakanalis A. (2017). Embodied medicine: Mens sana in corpore virtuale sano. Front. Hum. Neurosci..

[69] Park C.-B., Choi J.-S., Park S.M., Lee J.-Y., Jung H.Y., Seol J.-M., Hwang J.Y., Gwak A.R., Kwon J.S. (2014). Comparison of the effectiveness of virtual cue exposure therapy and cognitive behavioral therapy for nicotine dependence. Cyberpsychol. Behav. Soc. Netw..

[70] Kellmeyer P., Biller-Andorno N., Meynen G. (2019). Ethical tensions of virtual reality treatment in vulnerable patients. Nat. Med..

[71] Marloth M., Chandler J., Vogeley K. (2020). Psychiatric interventions in virtual reality: Why we need an ethical framework. Camb. Q. Healthc. Ethics.

[72] Weech S., Kenny S., Barnett-Cowan M. (2019). Presence and cybersickness in virtual reality are negatively related: A review. Front. Psychol..

[73] Bossuyt P.M., Reitsma J.B., Bruns D.E., Gatsonis C.A., Glasziou P.P., Irwig L., Lijmer J.G., Moher D., Rennie D., De Vet H.C. (2015). STARD 2015: An updated list of essential items for reporting diagnostic accuracy studies. Clin. Chem..

[74] Von Elm E., Altman D.G., Egger M., Pocock S.J., Gøtzsche P.C., Vandenbroucke J.P., Initiative S. (2014). The Strengthening the Reporting of Observational Studies in Epidemiology (STROBE) Statement: Guidelines for reporting observational studies. Int. J. Surg..

[75] Kim D.-Y., Lee J.-H. (2015). Development of a virtual approach–avoidance task to assess alcohol cravings. Cyberpsychol. Behav. Soc. Netw..

[76] Kim D.-Y., Lee J.-H. (2019). The Effects of Training to Reduce Automatic Action Tendencies Toward Alcohol Using the Virtual Alcohol Approach-Avoidance Task in Heavy Social Drinkers. Cyberpsychol. Behav. Soc. Netw..

[77] Papini S., Young C.C., Gebhardt C.S., Perrone A., Morikawa H., Otto M.W., Roache J.D., Smits J.A. (2020). Isradipine enhancement of virtual reality cue exposure for smoking cessation: Rationale and study protocol for a double-blind randomized controlled trial. Contemp. Clin. Trials.

[78] Machulska A., Eiler T.J., Grünewald A., Brück R., Jahn K., Niehaves B., Ullrich H., Klucken T. (2020). Promoting smoking abstinence in smokers willing to quit smoking through virtual reality-approach bias retraining: A study protocol for a randomized controlled trial. Trials.

[79] Mellentin A.I., Nielsen A.S., Ascone L., Wirtz J., Samochowiec J., Kucharska-Mazur J., Schadow F., Lebiecka Z., Skoneczny T., Mistarz N. (2020). A randomized controlled trial of a virtual reality based, approach-avoidance training program for alcohol use disorder: A study protocol. BMC Psychiatry.

[80] Chen X.J., Wang D.M., Zhou L.D., Winkler M., Pauli P., Sui N., Li Y.H. (2018). Mindfulness-based relapse prevention combined with virtual reality cue exposure for methamphetamine use disorder: Study protocol for a randomized controlled trial. Contemp. Clin. Trials.

[81] Liu W., Chen X.-J., Wen Y.-T., Winkler M.H., Paul P., He Y.-L., Wang L., Chen H.-X., Li Y.-H. (2020). Memory Retrieval-Extinction Combined With Virtual Reality Reducing Drug Craving for Methamphetamine: Study Protocol for a Randomized Controlled Trial. Front. Psychiatry.

