# Supplementary material for: Clinical Relevance of Immersive Virtual Reality in the Assessment and Treatment of Addictive Disorders: A Systematic Review and Future Perspective
Source: J Clin Med. 2021 Aug 18;10(16):3658. doi: 10.3390/jcm10163658 (PMC8396890; doi:10.3390/jcm10163658)
Supplement: Supplementary file 1 [file jcm-10-03658-s001.zip › jcm-1292383-supplementary.pdf]

**Table S1.** Definitions – VR therapy in Ads.

| **Concept** | **Description/Definition** |
|---|---|
| General – Virtual reality | |
| Virtual reality | Computer-generated simulation of a three-dimensional environment, which aims to immerse the user through the use of special electronic equipment [1]. |
| Virtual environment (VE) | VEs can be computer-generated, photorealistic images, or 360-degree videos of the real-world, which can be viewed within VR. HMDs allow to render the scenes relative to the persons position of the head and body movements. The interaction with objects and virtual agents is optional and dependent on the utilized VEs [2]. |
| Immersion | 'Immersion is a description of a technology, and described the extent to which the computer displays are capable of delivering an inclusive, extensive, surrounding and vivid illusion of reality to the senses of a human participant.' [3] (p. 3) |
| Presence | 'The subjective experience of being in one place or environment, even when one is physically situated in another.' [4] (p. 225) |
| Ecological validity | 'The degree to which the findings of research studies generalize to real-world settings.' [2] (p. 170) |
| Interactivity | 'Refers to the degree to which users of a medium can influence the form or content of the mediated environment.' [5] (p. 80) |
| Cybersickness | 'Cybersickness is comprised of a constellation of motion sickness-like symptoms which resemble true motion sickness. Symptoms may occur during VE system usage and often do not stop immediately upon post-exposure, but linger on, leaving those who are most affected feeling unsteady and disoriented.' [6] (p. 1138) |
| Technology | |
| Head-Mounted Display | 'Head-mounted displays consist of computer display screens in front of the eyes of the user, along with a head-tracking device. The head-tracking device provides head orientation and/or location orientation information to a computer that, in turn, shows the user in the HMD images that are consistent with the direction in which the user is looking within the virtual environment. The system may also provide spatial audio information to the user via stereo headphones.' [7] (p. 79) |
| CAVE- Cave Automatic Virtual Environment | 'The CAVE is a projection-based VR system. The illusion of immersion is created by projecting stereoscopic computer graphics into a cube composed of display-screens that completely surround the viewer. It is coupled with a head and hand tracking system to produce the correct stereo perspective and to isolate the position and orientation of a three-dimensional input device. A sound system provides audio feedback. The viewer explores the virtual world by moving around inside the cube and manipulating objects with a three-button wand-like device.' [8] (p. 59) |
| Sense of embodiment | 'SoE toward a body B is the sense that emerges when B's properties are processed as if they were the properties of one's own biological body.' [9] (p. 375) |
| Virtual agents | 'Virtual agents, animated interface agents, user interface agents, embodied conversational agents or virtual humans are computer generated characters that are capable of interacting with a user through the use of language. The simplest versions of these agents consist of various different images and some text as output. More advanced versions of these agents feature animated fully 3D-rendered bodies which allow an agent to have a natural, realistic look and |

| | |
|---|---|
| | communicate non-verbally through gestures and body language. These can speak with the user through speech synthesis and speech recognition.' [10] (p. 28) |
| **Cue-reactivity in VEs** | |
| Craving | Though a uniform definition is lacking, craving can be described as a 'pathological appetite' to use a certain substance, which is '… thought to reflect a drug acquisitive state motivating drug use' [11] (p. 2) |
| Psychophysiological response | 'These responses, controlled by the autonomic nervous system, are considered objective markers of cue-reactivity. Heart rate, skin conductance and temperature are the most studied psychological responses.' [12] (p. 4) |
| Attention to cue | 'Referring to attention bias, attention to cue is the motivational trend to focus on drug cues while neglecting or ignoring others type of stimuli.' [12] (p. 4) |
| **Cues in VEs** | |
| Proximal cues | 'This is the most frequent type of cue used in traditional cue-reactivity studies. Proximal cues are ubiquitous across drug use. They are more often visual cues such as cigarette, ashtray, lighters, bottle of alcohol but can also be olfactory, auditory and tactile.' [12] (p. 4) |
| Contextual (distal) cues | 'They refer to the environment or context, with or without social interaction, in which substance use occurs such as bar or party. As well as proximal cues and despite being less reliable, they can elicit conditioned responses by being previously paired with drug use.' [12] (p. 4) |
| Complex cues | 'A combination of proximal and contextual cues. They represent a more complete picture of real-world stimuli (people drinking alcohol in a party or smokers gathering outside a bar).' [12] (p. 4) |
| **VR treatment approaches** | |
| VRET | 'Typically, cue-exposure treatment involves repeated unreinforced exposure to stimuli associated previously with drug use in an attempt to extinguish an addict's conditioned responses to such cues.' [13] (p. 155) |
| (VR-)CBT | 'We define CBT as a time-limited, multi-session intervention that targets cognitive, affective, and environmental risks for substance use and provides training in coping skills to help an individual achieve and maintain abstinence or harm reduction.' [14] (p. 2) |
| (VR-)CC | 'The term counterconditioning refers both to the technique and the putative process by which behavior is modified through a new association with a stimulus of an opposite valence.' [15] (p. 1) |
| (VR-)CS | 'Covert sensitization is an imagery-based aversive treatment for decreasing craving and inducing aversion toward abused substances.' [16] (p. 1) |
| (VR-)AAT | 'Cognitive bias modification (CBM) is a growing area of addictions intervention research that aims to change maladaptive approach biases (i.e., automatic action tendencies to approach substance use cues; thereby diminishing use of the targeted substance (e.g., alcohol) and potentially changing neural activation in reward-related brain regions).' [17] (p. 196) |
| (VR-)EL | 'Embodied cognition defines cognition as an interaction between the mind and the body's systems. People generate mental representations through physical simulations, situated action, and bodily states. Grounded cognition and learning can occur at various levels of mental processing, taking into account abstract internal representations.' [18] (p. 223) |

## References


1. Steuer, J. Defining Virtual Reality: Dimensions Determining Telepresence, Department of Communication. *Stanford University, Cyborganic Media* **1993**, *67*, 94103-92214.
2. Bell, I.H.; Nicholas, J.; Alvarez-Jimenez, M.; Thompson, A.; Valmaggia, L. Virtual reality as a clinical tool in mental health research and practice. *Dialogues in clinical neuroscience* **2020**, *22*, 169.
3. Slater, M.; Wilbur, S. A framework for immersive virtual environments (FIVE): Speculations on the role of presence in virtual environments. *Presence: Teleoperators & Virtual Environments* **1997**, *6*, 603-616.
4. Witmer, B.G.; Singer, M.J. Measuring presence in virtual environments: A presence questionnaire. *Presence* **1998**, *7*, 225-240.
5. Steuer, J. Defining virtual reality: Dimensions determining telepresence. *Journal of communication* **1992**, *42*, 73-93.
6. Stanney, K.M.; Kennedy, R.S.; Drexler, J.M. Cybersickness is not simulator sickness. In Proceedings of the Proceedings of the Human Factors and Ergonomics Society annual meeting, 1997; pp. 1138-1142.
7. Anderson, P.L.; Rothbaum, B.O.; Hodges, L. Virtual reality: Using the virtual world to improve quality of life in the real world. *Bulletin of the Menninger Clinic* **2001**, *65*, 78-91.
8. Cruz-Neira, C.; Leigh, J.; Papka, M.; Barnes, C.; Cohen, S.M.; Das, S.; Engelmann, R.; Hudson, R.; Roy, T.; Siegel, L. Scientists in wonderland: A report on visualization applications in the CAVE virtual reality environment. In Proceedings of the Proceedings of 1993 IEEE Research Properties in Virtual Reality Symposium, 1993; pp. 59-66.
9. Kilteni, K.; Groten, R.; Slater, M. The sense of embodiment in virtual reality. *Presence: Teleoperators and Virtual Environments* **2012**, *21*, 373-387.
10. Klaassen, R. *HCI perspectives on behavior change support systems*; University of Twente: 2015.
11. Sayette, M.A. The role of craving in substance use disorders: Theoretical and methodological issues. *Annual review of clinical psychology* **2016**, *12*, 407-433.
12. Segawa, T.; Baudry, T.; Bourla, A.; Blanc, J.-V.; Peretti, C.-S.; Mouchabac, S.; Ferreri, F. Virtual reality (VR) in assessment and treatment of addictive disorders: A systematic review. *Frontiers in neuroscience* **2020**, *13*, 1409.
13. Conklin, C.A.; Tiffany, S.T. Applying extinction research and theory to cue - exposure addiction treatments. *Addiction* **2002**, *97*, 155-167.
14. Magill, M.; Ray, L.; Kiluk, B.; Hoadley, A.; Bernstein, M.; Tonigan, J.S.; Carroll, K. A meta-analysis of cognitive-behavioral therapy for alcohol or other drug use disorders: Treatment efficacy by contrast condition. *Journal of consulting and clinical psychology* **2019**, *87*, 1093.
15. Keller, N.E.; Hennings, A.C.; Dunsmoor, J.E. Behavioral and neural processes in counterconditioning: past and future directions. *Behaviour research and therapy* **2020**, *125*, 103532.
16. Choi, Y.J.; Lee, J.-H. The effect of virtual covert sensitization on reducing alcohol craving in heavy social drinkers. *Virtual Reality* **2015**, *19*, 111-117.
17. Jacobus, J.; Taylor, C.T.; Gray, K.M.; Meredith, L.R.; Porter, A.M.; Li, I.; Castro, N.; Squeglia, L.M. A multi-site proof-of-concept investigation of computerized approach-avoidance training in adolescent cannabis users. *Drug and alcohol dependence* **2018**, *187*, 195-204.
18. Bailey, J.O.; Bailenson, J.N.; Casasanto, D. When does virtual embodiment change our minds? *Presence: Teleoperators and Virtual Environments* **2016**, *25*, 222-233.
